# Pathogenesis and Management in Cerebrovenous Outflow Disorders

**DOI:** 10.14336/AD.2020.0404

**Published:** 2021-02-01

**Authors:** Chaobo Bai, Zhongao Wang, Christopher Stone, Da Zhou, Jiayue Ding, Yuchuan Ding, Xunming Ji, Ran Meng

**Affiliations:** ^1^Department of Neurology, Xuanwu Hospital, Capital Medical University, Beijing, China.; ^2^Advanced Center of Stroke, Beijing Institute for Brain Disorders, Beijing, China.; ^3^Department of China-America Institute of Neuroscience, Xuanwu Hospital, Capital Medical University, Beijing, China.; ^4^Department of Neurosurgery, Wayne State University School of Medicine, Detroit, Michigan, USA.; ^5^Department of Neurosurgery, Xuanwu Hospital, Capital Medical University, Beijing, China

**Keywords:** cerebral venous thrombosis, cerebral venous sinus stenosis, internal jugular vein stenosis, diagnosis, treatment

## Abstract

In keeping with its status as one of the major causes of disability and mortality worldwide, brain damage induced by cerebral arterial disease has been the subject of several decades of scientific investigation, which has resulted in a vastly improved understanding of its pathogenesis. Brain injury mediated by venous etiologies, however, such as cerebral, jugular, and vertebral venous outflow disturbance, have been largely ignored by clinicians. Unfortunately, this inattention is not proportional to the severity of cerebral venous diseases, as the impact they exact on the quality of life of affected patients may be no less than that of arterial diseases. This is evident in disease sequelae such as cerebral venous thrombosis (CVT)-mediated visual impairment, epilepsy, and intracranial hypertension; and the long-term unbearable head noise, tinnitus, headache, dizziness, sleeping disorder, and even severe intracranial hypertension induced by non-thrombotic cerebral venous sinus (CVS) stenosis and/or internal jugular venous (IJV) stenosis. In addition, the vertebral venous system (VVS), a large volume, valveless vascular network that stretches from the brain to the pelvis, provides a conduit for diffuse transmission of tumors, infections, or emboli, with potentially devastating clinical consequences. Moreover, the lack of specific features and focal neurologic signs seen with arterial etiologies render cerebral venous disease prone to both to misdiagnoses and missed diagnoses. It is therefore imperative that awareness be raised, and that as comprehensive an understanding as possible of these issues be cultivated. In this review, we attempt to facilitate these goals by systematically summarizing recent advances in the diagnosis and treatment of these entities, including CVT, CVS stenosis, and IJV stenosis, with the aim of providing a valid, practical reference for clinicians.

In recent years, long-term brain dysfunction caused by cerebral and internal jugular venous outflow disorders has become increasingly well-understood. Despite this, it has not received attention in proportion to its elucidation, as has been the case for cerebral arterial diseases. Experimental and clinical research investigating cerebral and internal jugular venous outflow disturbance and other related entities is consequently still limited in comparison with arterial etiologies. Nevertheless, the impact exacted on the quality of life of affected patients may be no less than that of arterial diseases. Examples of severe sequelae of venous disorders abound, including CVT-mediated visual impairment, epilepsy, and intracranial hypertension [1-5]; and non-thrombotic CVS stenosis and/or IJV stenosis-induced long-term unbearable head noise, tinnitus, headache, dizziness, sleeping disorder, and severe intracranial hypertension [6-20].

In addition, the vertebral venous system (VVS), a large-volume, valveless vascular network that stretches from the brain to the pelvis and facilitates bilateral communication into and throughout the central nervous system, provides a conduit for diffuse transmission of tumors, infections, or emboli, with potentially devastating clinical consequences. Moreover, the lack of specific features and focal neurologic signs seen with arterial etiologies render cerebral venous disease prone to both to misdiagnoses and missed diagnoses. Whereby, it is imperative to raise awareness and make a comprehensive understanding of these issues.

In this review, we attempt to facilitate these goals by systematically summarizing recent advances in the diagnosis and treatment of these entities, including CVT, CVS stenosis, and IJV stenosis, with the aim of providing a valid, practical reference for clinicians.

## 1.Cerebral venous thrombosis

### 1.1 Etiology

Cerebral venous thrombosis (CVT) is a subtype of stroke that mainly affects young individuals. The pathological mechanism of CVT has not yet been fully elucidated, but vessel wall dysfunction, alterations in hemodynamics, and changes in blood composition are all recognized as potential contributors. A variety of risk factors may predispose patients to CVT; risk factors are classified as either non-genetic, as in pregnancy and malignancy, or genetic, as in the hereditary thrombophilias.

### 1.1.1Non-genetic risk factors

#### Pregnancy and the postpartum period

Both pregnancy and the postpartum period have been widely considered to be common risk factors for CVT; however, recent studies have shown that a higher risk of CVT is associated with the postpartum period, and that pregnancy is not as significant. A multicenter, case-control study from the Netherlands in 2019, for instance, indicated that pregnancy did not increase the risk of CVT compared with the control group, while the risk of CVT was increased 10-fold during the postpartum period [21]. In addition, a large, multicenter clinical study (VENOST study) in Turkey involving 1144 patients in 2017 showed both that gynecologic risk factors constituted the largest group of causes of CVT, and that the postpartum period was the most prevalent of all gynecologic risk factors [22]. A real-world cohort study from China yielded similar results, showing that the top risk factor for CVT in females was the postpartum period [23].

#### Oral contraceptive use

Oral contraceptive use is also a common risk factor for CVT. A meta-analysis by Dentali F et al. suggested that patients with oral contraceptives had a high risk of developing CVT (odds ratio (OR) 5.59; 95% confidence interval [CI] 3.95 to 7.91; *p*<0.001) [24]. These results were replicated in another meta-analysis conducted by Amoozegar F et al., which found that women aged 15-50 who had taken oral contraceptives had a CVT risk 7.59 times higher than that possessed by women not taking oral contraceptives. (OR = 7.59, 95% CI 3.82-15.09) [25].

#### Tumors

As CVT is essentially a deep vein thrombosis (DVT) that impacts the cerebral circulation, it is not surprising that it shares with DVT an association with tumors, especially when the tumors are malignant. Potential mechanisms underlying this association include direct compression, invasion of the cerebral venous sinus by malignant tumors, and malignant tumor-mediated hypercoagulable states [1].

Strong clinical evidence has confirmed that tumors in general, and hematological malignancies in particular, are risk factors for CVT. The findings from a case-control study by Silvis SM et al. involving 594 patients with CVT and 6278 controls suggested that patients with tumors had a 5-fold higher risk of CVT. Notably, the risk of CVT reached nearly 90-fold within 1 year after the diagnosis of hematological malignancies [26].

#### Obesity

A case-control study from the Netherlands in 2016 reported that obesity is significantly associated with CVT in women. Obese women were nearly 3.5 times more likely to develop CVT than non-obese patients, and the use of oral contraceptives further increased the risk of overweight and obese women by 11 and 29 times, respectively [27].

#### Other risk factors

Other risk factors, including systemic immune diseases, brain trauma, arteriovenous malformations, infections, head and neck surgery, spontaneous low intracranial pressure, anemia, dehydration, thyroid dysfunction, and hyperhomocysteinemia, etc., all may also contribute to the occurrence of CVT [28, 29].

### 1.1.2Genetic thrombophilia

#### Anti-thrombin, protein C, and protein S deficiencies

Screening for genetic thrombophilia is an integral part of the CVT risk factor assessment. A number of common etiologies have been described, including deficiencies of anti-thrombin, protein C, and protein S [30]. The results of Martinelli et al. indicated that the prevalence of anti-thrombin, protein C, and protein S deficiency in patients with CVT are 2.5, 5.2, and 3.1%, respectively [31]. Two high-quality studies investigated the role of anti-thrombin, protein C, and protein S deficiency as risk factors for CVT. The combined ORs of these studies were 2.69 (95% CI, 0.66-10.96; *p*<0.19) for anti-thrombin, 11.10 (95% CI, 1.87-66.05; *p*<0.009) for protein C deficiency, and 12.49 for protein S deficiency (95% CI, 1.45-107.29; *p*<0.03) [24, 28-30].

#### Prothrombin G20210A and factor V Leiden

Prothrombin G20210A and factor V Leiden are regarded as common risk factors for arterial ischemic stroke as well as CVT [32]. In a meta-analysis conducted by Green M et al. in 2018, however, it was reported that Prothrombin G20210A and factor V Leiden polymorphisms had a greater effect on CVT than on arterial ischemic stroke [33]. In addition, the meta-analysis results reported by Dentali F et al. suggested that factor V Leiden (OR 3.38; 95% CI 2.27 to 5.05; *p*<0.001) combined with the prothrombin G20210A mutation (OR 9.27; 95% CI 5.85 to 14.67; *p*<0.001) significantly increases the risk of CVT [24].

#### FVIII

A matched case-control study in 2018 that included 116 patients and 116 controls by Vecht L et al. reported that elevated FVIII was frequently observed in CVT patients (CVT vs. control was 83.6% vs. 28.4%, *p*<0.001). Patients with elevated FVIII had a nearly 15-fold increased risk of developing CVT (OR 15.3, 95% CI 7.8-30.1) compared with controls. Interestingly, the association between FVIII and CVT was more prominent in male patients than in females [34].

#### FXII

In 2008, Reuner KH et al. analyzed 78 consecutive patients with CVT and 201 healthy members of the general population of southern Germany, finding that the TT genotype of the FXII C46T gene may be a new independent risk factor for CVT (OR 4.57; 95% CI 1.55-13.41; *p*=0.006) [35]. Similarly, a study by Prabhakar P et al. from India in 2012 showed that this FXII mutation disrupts the vital role played by FXII in the fibrinolytic pathway, resulting in a 2.9-fold increase in the risk of CVT [36].

#### JAK2 V617F mutation

There is currently controversy over the advisability of routine JAK2 gene screenings for CVT patients. A study from France in 2008 found that the likelihood of a positive screen for JAK2 mutations in CVT patients without myeloproliferative disorders was as low as 1.1%, and therefore concluded that routine testing is not recommended [37]. However, during the same period, an Italian study found a higher mutation rate of about 6.2%, leading the authors to suggest that JAK2 mutations should be sought in the interest of developing a more rational early management protocol for patients carrying the mutation [38]. Another retrospective cohort study from Italy in 2012 agreed, finding a 6.6% mutation rate and proposing that the JAK2 V617F mutation should be considered as a screening option [39].

### 1.2 Clinical subtypes

#### 1.2.1 Cerebral venous sinus thrombosis (CVST)

Cerebral venous sinuses include the superior sagittal sinus, inferior sinus, transverse sinus, straight sinus, sigmoid sinus, and cavernous sinus. CVST is the most common subtype of CVT, the clinical manifestations of which include headache, visual impairment, epilepsy, focal neurological deficits, and altered consciousness.

#### 1.2.2 Cortical venous thrombosis

Isolated cortical venous thrombosis is not common and has only been reported in individual cases. The clinical manifestations of isolated cortical venous thrombosis are similar to those of venous sinus thrombosis, either alone or in combination with other symptoms such as papilledema, epilepsy, and focal neurological deficits. Of these symptoms, it has been reported in the literature that focal neurological impairment and epilepsy were more common in patients with isolated cortical venous thrombosis [40-42].

#### 1.2.3 Cerebral deep venous thrombosis

Cerebral deep venous thrombosis is a relatively rare and potentially lethal subtype of CVT. The vein of Galen and the basal vein are the principal conduits of deep venous outflow responsible for drainage of the cerebral hemispheric white matter, basal ganglia, thalamus, and diencephalon. Because cerebral deep venous thrombosis affects venous outflow in this territory, it may cause mental status alterations, gaze palsy, and diffuse encephalopathy or coma [43-46].

### 1.3 Clinical manifestations

#### 1.3.1 Headache

The symptoms with which CVT presents are highly variable between individuals. Headache is usually the first, and is also the most common symptom of CVT [47, 48]. It frequently accompanies other clinical symptoms but may also present in isolation, and only 10% of CVT patients do not report it [49]. In CVT, most headaches present acutely or sub-acutely; only a few are chronic. The location of the headache has no significant correlation with the lesion, unless the lateral sinus is involved, in which case patients are prone to neck or occipital pain [50]. The properties of CVT headaches are diverse, including thunderclap, migraine-like, cluster, post-dural puncture, band-like, and others [2]. This headache variability and lack of specificity can result in diagnostic delay. Therefore, clinicians should be aware that some types, such as aggravated headaches, thunderclap-like headaches, or headaches while lying down or during the Valsalva maneuver, should prompt concern for CVT [51, 52].

#### 1.3.2 Seizure

Epileptic seizures are a feature of the presentation in nearly 40% of severely acute CVT cases, a higher rate than that seen in either acute arterial stroke or intracranial hemorrhage [48, 53]. Seizures may be generalized or focal, and some patients may experience both. Patients with epilepsy generally manifest sensorimotor deficits, hemorrhagic infarcts, frontal lobe lesions, or superior sagittal sinus or cortical venous thrombosis [3, 54, 55]. Status epilepticus, which is considered a neurological emergency, occurs in 5.6%-7% of CVT [3, 55]. The impact of epilepsy on CVT mortality is still controversial. It was reported that status epilepticus is one of the factors leading to the death in CVT patients, and that mortality with status epilepticus is three times higher than that seen with non-sustained seizures [55]. On the other hand, findings from another study suggested that there is no significant association between seizures and mortality or 90-day prognosis [56]. Still, it is worth noting that early identification and management of epilepsy may help minimize neurological impairment.

#### 1.3.3 Focal neurological deficits

About 40% of CVT patients present with focal neurological deficits, including motor or sensory impairment, aphasia, and cranial nerve palsies [57]. In CVT, brain parenchymal impairment may result from insufficient compensatory venous outflow, precipitating focal neurological deficits affecting the obstructed region. Because of the extensive anastomoses and physiological variation that characterize the intracranial veins, however, the connection between focal neurological deficits and impaired vessels may be inconsistent. Unlike in ischemic stroke, most of these deficits are caused by vasogenic edema secondary to blood-brain barrier impairment, which resolves relatively readily [58]. Focal deficits are more common in non-inflammatory CVT, while cavernous sinus syndrome is more commonly observed in infection-associated CVT.

#### 1.3.4 Altered consciousness

Approximately 14% of CVT cases feature severely altered consciousness in the early phase [1]. The presence of altered consciousness in CVT is usually thought to be associated with involvement of the deep venous system [59]. Although CVT patients with altered consciousness are more likely to get an early diagnosis, coma is a strong predictor of poor prognosis: it is reported that nearly 25% of patients still have progressive coma despite aggressive anticoagulation [60]. Therefore, the establishment of a rapid diagnostic protocol and more effective intervention strategies for patients with severely altered consciousness are priorities in the effort to improve the prognosis of severe CVT.

#### 1.3.5 Visual manifestations

CVT can cause neuro-ophthalmological symptoms, including visual impairment and papilledema. Visual impairment, including vision loss and visual field defects, may be linked pathogenically to papilledema induced by elevated intracranial pressure or focal lesions. Papilledema can be found in most CVT patients, suggesting the presence of increased intracranial pressure. Fortunately, the majority of visual impairment induced by CVT is restorable, although persistent papilledema may ultimately cause permanent optic atrophy. In addition, it has been reported that CVT can manifest as psychiatric symptoms and transient global amnesia [1, 2, 61-64].

### 1.4 Diagnosis

#### 1.4.1 CT/ CT venography (CTV)

CT is the most commonly used initial examination tool for sudden, new-onset presentations suggesting CVT, such as headache, epilepsy, changes in consciousness, and focal neurological signs. However, only about one-third of patients exhibit the direct CVT signs of high attenuation in the cortical veins or cerebral venous sinuses. Indirect signs, such as cerebral hemorrhage and subarachnoid hemorrhage, occur infrequently. CVT may also appear as a filling defect of the cerebral venous sinus on contrast-enhanced CT [65, 66].

CTV can provide fast and reliable results, exhibiting a diagnostic efficacy at least equal to that of MRV. The main drawbacks of this modality are the radiation exposure it entails and the limitations of its application imposed by allergies to iodinated contrast material [53, 67, 68].

#### 1.4.2 MRI/Time-of-Flight (TOF)-MRV/Contrast-enhanced (CE)-MRV/Phase Contrast (PC)-MRV

**Figure 1.**
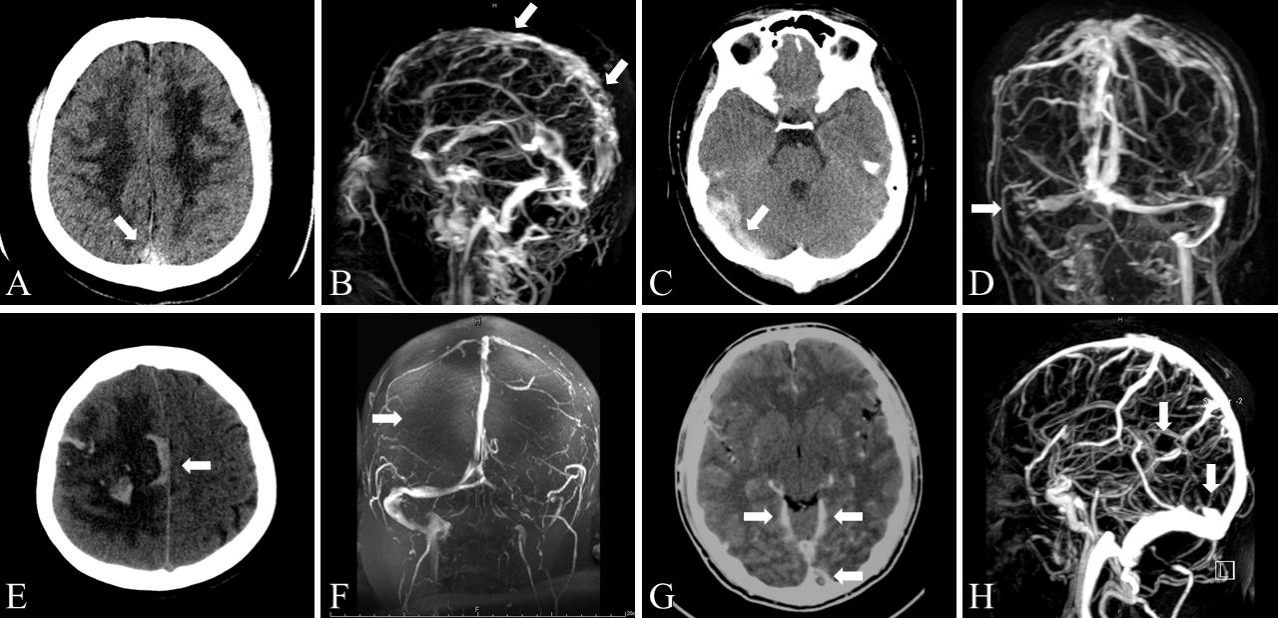
Imaging findings using CT and MRV in patients with CVT. (A-B) Superior sagittal sinus thrombosis. Axial CT demonstrated hyperintense signal intensity of the superior sagittal sinus (A). Sagittal CE-MRV confirmed thrombosis in the superior sagittal sinus (B). (C-D) Right transverse sinus and sigmoid sinus thrombosis. Axial CT demonstrated hyperintense signal intensity of the right transverse sinus (C). MRV confirmed thrombosis in the right transverse sinus and sigmoid sinus (D). (E-F) Cortical venous thrombosis. Axial CT scan of a patient with cortical venous thrombosis showed a venous hemorrhagic infarct in the right parietal lobe (E). TOF-MRV showed disappearance of the right cortical veins. The white arrow indicates that this right cortical venous disappearance was caused by a thrombus (F). (G-H) Cerebral deep venous thrombosis. Hyperintense signal intensity of the straight sinus and bilateral deep cerebral veins was observed using axial CT (G). With sagittal CE-MRV, the straight sinus and bilateral deep cerebral veins were not observed. The white arrows indicate filling defects that were caused by the thrombus (H).

MRI and MRV, owing to the high accuracy they confer, have gradually become standard diagnostic tools for CVT [69]. The primary problem posed by their use is a scanning speed that is inadequate to meet the demand faced by the emergency department. Another issue concerns the inferiority of MRV to DSA in diagnostic accuracy for stenosis, due to which it may generate false-positives and false-negatives for this entity [70]. Finally, while conventional MR sequences are impaired by limited resolution for solitary cortical venous thrombosis, advances in MRI scanning and imaging technology have mitigated this concern. For instance, while 2D-TOF-MRV is the most commonly applied tool for diagnosing CVT in clinical practice, its diagnostic accuracy is inferior to that of 3D-CE-MRV, which provides better image quality and resolution of intracranial veins, as well as superior inter-operator consistency due to a short scanning time. However, it is worth noting that CE-MRV is prone to false-negative results in chronic thrombosis or in cases of partially recanalized thrombi[71], and that contrast injection and the presence of poor renal function limit its application [72]. Another promising novel imaging modality is 3D-PC-MRV, which offers the advantage of flow quantification to assist in distinguishing between thrombi and venous blood flow. 3D-PC-MRV also provides a realistic velocity map that supports the assessment of venous flow and the degree of stenosis. The disadvantages of this method relate to the long scanning time it requires, and the ideal velocity encoding that may generate motion artifacts and inconsistencies between operators [73] (Fig. 1).

#### 1.4.3 Invasive diagnostic procedures

##### Cerebral angiography

Cerebral angiography is generally appropriate when the diagnosis cannot be determined after MRV and CTV, or when an endovascular treatment procedure is under consideration. Aplasia or malformations of cerebral veins or venous sinuses can be clarified during the venous phase of cerebral angiography. If a cerebral vein or venous sinus is not visualized with angiography, the suspicion for acute CVT should be high [74, 75].

##### Cerebral venography

Direct cerebral venography involves injection of contrast material using micro-catheters into the cerebral vein or venous sinus after introduction through the internal jugular vein. When this is done, CVT with partial obstruction manifests as a filling defect, while fully obstructed venous vessels show no filling. Direct cerebral venography is also capable of measuring venous pressure, rendering it useful in cases when intracranial venous hypertension is suspected [74, 75].

**Figure 2.**
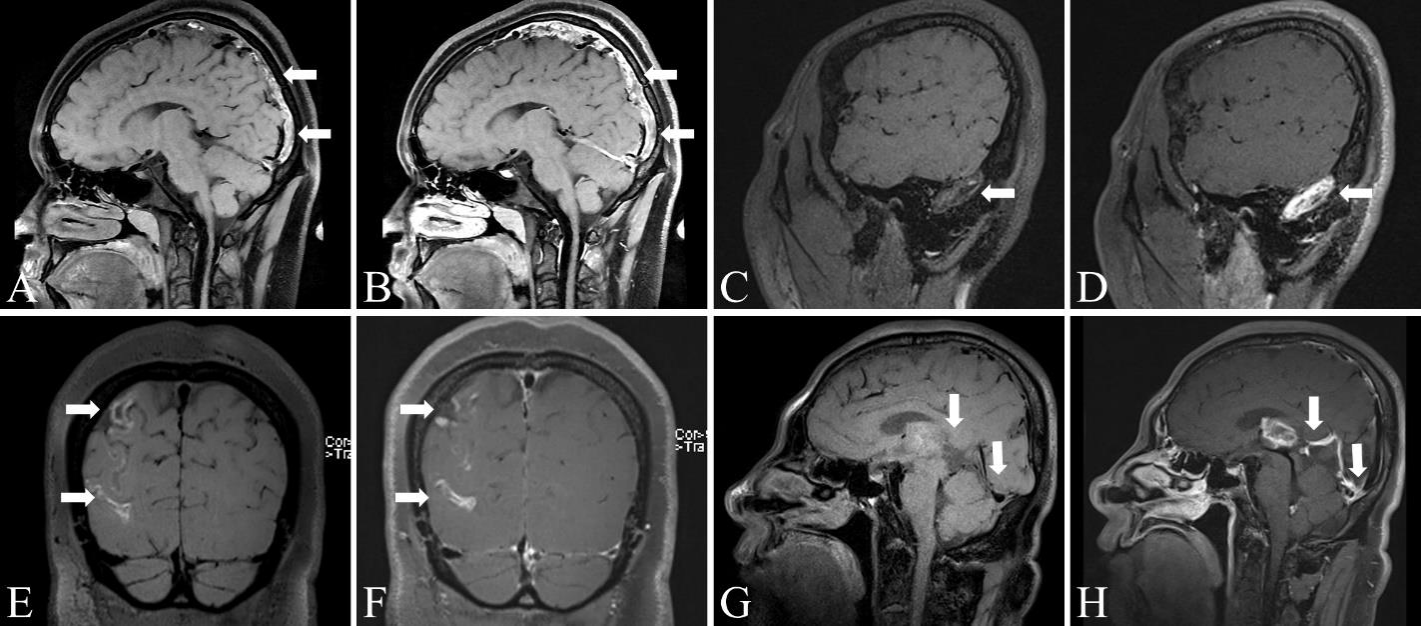
Imaging findings using MRBTI in patients with CVT. (A-B) Subacute superior sagittal sinus thrombosis. Sagittal MRBTI and MRBTI enhancement showed significant hyperintense signal in the superior sagittal sinus, suggesting intraluminal subacute thrombus formation. (C-D) Chronic right transverse sinus and sigmoid sinus thrombus. Sagittal MRBTI showed significant isointense and hyperintense signal in the right transverse sinus and sigmoid sinus, suggesting intraluminal chronic thrombus formation (C). Sagittal MRBTI enhancement showed that the lumen in the right transverse sinus and sigmoid sinus was enhanced. The white arrows indicate that new blood vessels appeared inside the thrombus (D). (E-F) Subacute cortical venous thrombosis. Coronal MRBTI and MRBTI enhancement demonstrated hyperintense signal in the cortical veins of the right parietal and occipital lobes, suggesting subacute intraluminal thrombus formation. (G-H) Chronic straight sinus thrombosis. Sagittal MRBTI showed significant isointense and hypointense signal in the straight sinus and the deep cerebral veins (G). Sagittal MRBTI enhancement showed that the lumen in the straight sinus and the deep cerebral veins was enhanced. The white arrows indicate that new blood vessels appeared inside the thrombus (H).

#### 1.4.4 Advanced diagnostic modalities

##### Transcranial Doppler

Transcranial Doppler may support the diagnosis of CVT and is useful for its capacity to detect the venous blood flow velocity and hemodynamic pattern. Its main drawback is susceptibility to interference from bony structures [76, 77].

##### 4D flow MRI

4D-flow-MRI allows visualization and quantification of physiological cerebral venous hemodynamics; the quantitative analysis of cerebral venous blood flow by 4D-flow-MRV demonstrates high reliability and accuracy. 4D-flow-MRV is limited by an imperfect ability to visualize cerebral venules [78].

##### T2*gradient-echo sequence

Although MRV supplies high resolution when tasked with imaging major venous thromboses, the T2*gradient-echo sequence demonstrates a diagnostic value superior to traditional MRV for the variable, superficial cortical veins [79].

##### Magnetic resonance black-blood thrombus imaging technique (MRBTI)

Traditional imaging techniques detect thrombi primarily indirectly, through changes in venous blood flow. This renders them susceptible to interference, resulting in a mismatch between the image and the actual thrombus. In 2016, Yang Q et al. established MRBTI as a potential solution to this problem, and thus as a potential first-line diagnostic tool for CVT. MRBTI can effectively suppress the blood flow signal to black in order to directly detect the thrombus itself, which then appears with high signal intensity. Furthermore, MRBTI can quantify the volume of thrombus using software assistance. Accordingly, the occurrence of false-positives may be diminished compared to conventional MRV, thereby improving the accuracy of CVT detection [80]. In addition, this sequence has been shown to yield good sensitivity in both acute and non-acute CVT patients, thus contributing value both to the early identification of CVT and further observation over time [81]. MRBTI does not require contrast agents, and its capacity to directly quantify volume variation of the thrombus renders it useful for CVT follow-up. Despite these promising features, however, larger and multicenter studies are required to generate more evidence to validate the application of this emerging technology for use in CVT (Fig. 2).

#### 1.4.5 D-Dimer and NSE measurement

Measurement of the fibrin degradation product D-dimer can be performed easily in the emergency room, exhibits high sensitivity for the initial diagnosis of CVT, and is also, given its high negative predictive value, a reliable way to exclude it. Normal D-dimer levels measured by sensitive immunoassay or rapid ELISA predict a low probability of CVT [82-84]. However, several studies have offered evidence that negative D-dimers may have a limited efficacy for ruling out CVT in the context of a recently isolated headache [85, 86].

Serum neuron-specific enolase (NSE), located in neurons and neuroendocrine-derived cells, is a sensitive laboratory biomarker for brain injury. Although there is currently little research designed to evaluate the value of NSE levels in the workup of CVT, a recent retrospective study found that baseline NSE levels were associated with CVT severity, and had the potential to predict prognosis [87].

### 1.5 Treatment

#### 1.5.1 Anticoagulation

Anticoagulation is the first-line therapy for initial CVT treatment. It may be achieved using traditional anticoagulants such as unfractionated heparin, low molecular weight heparin, and warfarin; as well as with new anticoagulants, such as dabigatran and rivaroxaban. Anticoagulant therapy functions by halting the growth of existing thrombi, promoting recanalization, and preventing deep vein thrombosis and pulmonary embolism. The new anticoagulants offer the advantages of requiring infrequent blood testing and conferring low risks of thrombus recurrence and bleeding, while their main drawback has historically been the unavailability of antagonists; this has recently changed, however, with the advent of the reversal agents idarucizumab and andexanet alfa [88-91].

#### 1.5.2 Fibrinolytic Therapy

The rate of partial or complete recanalization following anticoagulant therapy alone has been estimated to fall between 47%-100%, and 9%-13% of CVT has been shown to carry a poor clinical prognosis despite sufficient anticoagulant therapy. Thus, simple anticoagulation may insufficient for some patients, particularly when large and extensive thromboses are present. In these cases, fibrinolytic therapy may be added as a supplementary treatment. Fibrinolytic therapy may be delivered non-invasively with novel drug therapies such as batroxobin, or using invasive therapeutic strategies such as direct catheter thrombolysis and mechanical thrombectomy /thrombolysis [53].

Batroxobin is a non-invasive drug therapy that may promote CVT recanalization. Ding et al. analyzed the safety and efficacy of batroxobin in combination with anticoagulants on CVT and found a higher recanalization rate in the batroxobin group compared with the control group, especially in CVT patients with a high level of fibrinogen. Severe hemorrhagic events were not observed [92, 93].

Endovascular therapy may be considered when disease exhibits rapid progression, resulting in sequelae such as severe neurological impairment or altered consciousness [94]. Early recanalization of CVT generally predicts a better prognosis [95]. Regardless of drug therapy or endovascular treatment, however, it is difficult to achieve ideal recanalization in some patients. In 2018, a study from the United States assessed these refractory patients, showing that, in CVT patients with a poor response to endovascular therapy, using "microcatheter contact with alteplase perfusion" may effectively achieve recanalization without adverse outcomes, rendering this a promising alternative to be pursued after failure of standard treatment [96]. Currently, prioritization between the various endovascular methods for CVT therapy cannot be conducted due to insufficient clinical evidence [97].

#### 1.5.3 Surgery

Given the availability of both standardized anticoagulation and endovascular therapies for CVT, surgical treatment plays a limited role. Life-saving surgical interventions such as decompressive craniotomy should be considered, however, if the patient's neurological symptoms continue to deteriorate or if imaging confirms severe brain tissue compression despite maximal non-surgical treatment [98-101].

## 2. Non-thrombotic cerebral venous sinus (CVS) stenosis

### 2.1 Proposed etiology

Although neither the origin nor the pathogenesis of non-thrombotic CVS stenosis is well-defined, a number of factors are believed to contribute to its onset. These etiologies are classified as either extrinsic or intrinsic.

#### 2.1.1 Extrinsic anomalies

Broadly speaking, any extraluminal anomalies that impinge on the venous wall may cause CVS stenosis. Due to the present lack of research designed to investigate CVS stenosis, current etiologic theory is mainly derived from clinical experience. A few case reports have produced evidence for extrinsic impingement by describing an association between meningiomas and CVS stenosis. In such cases, an intracranial mass occupying the vicinity of the intracranial venous sinuses may directly invade the venous walls, leading to venous stenosis and outflow obstruction. If involvement of the cerebral venous sinuses is sufficiency extensive, this can cause severe, life-threatening cerebral edema [102-105].

Another potential, though controversial, impingement-based etiology is intracranial hypertension. Compelling evidence has been produced for a close association between isolated intracranial hypertension and transverse sinus stenosis: elevated intracranial hypertension may cause the collapse of cerebral venous sinus walls, resulting in CVS stenosis and outflow obstruction [11, 106, 107]. Nevertheless, Peng et al. proposed that the effects of intracranial hypertension and CVS stenosis may, rather than the former giving rise to the latter, be better conceived as mutually reinforcing [61].

#### 2.1.2 Intrinsic anomalies

The other category of CVS stenosis etiologies is intrinsic anomalies, which refers to proposed intraluminal causes. Arachnoid granulations represent this category. These are normal intracranial structures that protrude into the dural sinus lumen and function to facilitate drainage of cerebrospinal fluid into the cerebral venous system. Generally, arachnoid granulations larger than 1cm are considered giant, and may appear as local dilatations or filling defects within the cerebral venous sinus. Most giant arachnoid granulations are discovered incidentally and only rarely cause clinical symptoms, but may narrow or obstruct the lumen of the dural sinuses, leading to intracranial venous hypertension [108-110]. Notably, multiple consecutive giant arachnoid granulations appear similar to CVT on MRV, and may therefore be misdiagnosed as such [111].

### 2.2 Clinical manifestations

#### 2.2.1 Intracranial hypertension

Isolated intracranial hypertension is a syndrome characterized by elevated intracranial pressure of an uncertain etiology. Various etiological hypotheses have been proposed, including intracranial venous stenosis, increased secretion or decreased absorption of cerebrospinal fluid, and the effects of hormones [61]. Nevertheless, despite evidence that intracranial sinus stenosis is closely related to intracranial hypertension, the causal relationship between these entities remains, as mentioned above, uncertain [106]. It has been shown, however, that intracranial venous stenting was effective in relieving medically refractory isolated intracranial hypertension, suggesting that intracranial venous outflow disorders may promote the development of intracranial hypertension [6-10]. The common intracranial hypertension-related clinical manifestations such as headache, visual impairment, and papilledema may occur in the setting of CVS stenosis, but vary among individuals [11-13].

#### 2.2.2 Tinnitus

Unilateral or bilateral pulsatile tinnitus is another common symptom of CVS stenosis. When it presents in isolation most patients tolerate this symptom, resulting in treatment delays or misdiagnoses due to the lack of accompanying focal neurological signs. Accordingly, pulsatile tinnitus may last for many years before the affected patient is finally diagnosed with CVS stenosis. Although the mechanism of tinnitus in this context is unknown, it has been shown to respond exceptionally well to endovascular stenting, which may imply an etiologic relationship to changes in intracranial venous blood flow [14-20].

### 2.3 Diagnosis

#### 2.3.1 MRV

MRV is a widely accepted, non-invasive method for assessing the cerebral venous system. It has several advantages, including avoidance of ionizing radiation exposure, no need to use a contrast agent, non-invasiveness, and suitability for long-term follow-up and pregnant women. One area in which MRV falls short is in the display of veins with slow flow velocity [67, 112].

Time-of-Flight magnetic resonance venography (TOF-MRV) is the most used modality for the diagnosis of CVS stenosis, and the two-dimensional TOF MRV has good sensitivity compared with three-dimensional TOF MRV for demonstrating slow flow. Disadvantages of TOF MRV include a limited capacity to display intracranial venules, as well as susceptibility to interference from tissue signals (e.g., from fat), resulting in errors in image interpretation [43, 79, 113-118].

Compared to TOF-MRV, CE-MRV enhances visualization of the intracranial venous system. This visual superiority comes at the expense of requiring the use of a contrast agent, however, rendering CE-MRV unsuitable for patients who are allergic to the contrast agent and for pregnant women.

**Figure 3.**
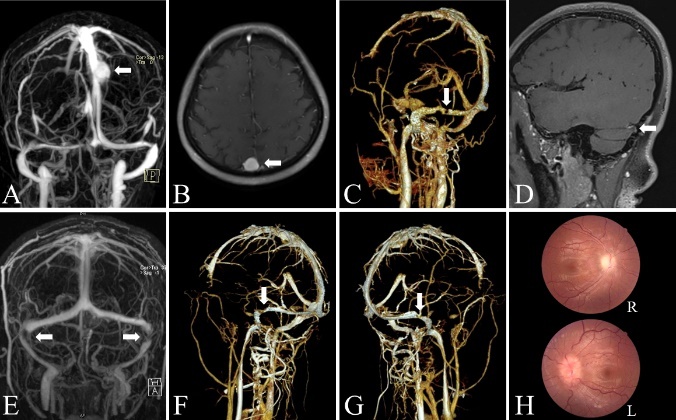
Imaging findings in patients with CVS stenosis. (A-B) Large meningioma-induced superior sagittal sinus stenosis seen with CE-MRV and with MRI enhancement. In CE-MRV, a well-defined meningioma caused confined stenosis of the superior sagittal sinus (A). The meningioma demonstrated hyperintense signal intensity with MRI enhancement, and caused significant compression of the superior sagittal sinus (B). (C-D) Large arachnoid granulation-induced transverse sinus stenosis seen with CTV and MRBTI. Severe stenosis of the left transverse sinus was observed with CTV (C). MRBTI showed a large arachnoid granulation in the left transverse sinus (D). (E-H) Severe bilateral transverse sinus stenosis seen with MRV and CTV. Severe stenosis of the bilateral transverse sinuses was observed using MRV (E) and CTV (F-G). Fundus examination showed bilateral papilledema (Frisen grade Ⅳ in the left eye and Frisen grade II in the right eye) (H).

#### 2.3.1 CT venography (CTV)

The term “CT-venography” was first described by Casey et al. as a technique capable of rapid generation of high-quality images of the cerebral venous circulation [119]. CTV is a non-invasive and relatively inexpensive detection method characterized by fast scanning time and convenient operation. For patients with ferromagnetic implants or cardiac pacemakers on the body, it is more suitable than MRV. In addition, the immediacy with which CTV can be carried out makes it superior to MRV in the management of critically ill patients.

In a study involving 24 patients, CTV was shown to be superior to MRV in showing the details of small veins, and was more reliable in revealing the basal veins, the thalamostriate veins, and the inferior sagittal sinus [120]. These researchers then concluded that the diagnostic value of CTV is equal to that of MRV. Similarly, Casey et al. showed that CTV is preferable to MRV in displaying vein details and venous collateral circulation. The use of CTV has been limited, however, by the exposure to ionizing radiation and injection of contrast agents it entails, and its unsuitability for pregnant women or repeated follow-up [67, 121, 122] (Fig. 3).

#### 2.3.2 Digital subtraction angiography (DSA) and Direct Cerebral Venography (CV)

DSA is well-known as the gold standard for evaluating intracranial venous anatomy, showing features such as hypoplasia or atresia of the intracranial veins and dural sinuses, and is also capable of providing detailed information on the flow dynamics of contrast media. CV is invariably performed during endovascular therapy and is accomplished first by insertion of microcatheters directly into the internal jugular vein, after which contrast media is injected into the dural sinus or cerebral vein. Because these methods are invasive and require exposure to radiation and contrast media, both are considered suboptimal diagnostic techniques [74, 75, 124].

#### 2.3.3 Transcranial Doppler (TCD)/Transcranial color-coded duplex sonography (TCCS)

Ultrasound examination of CVS is a technique developed in recent years that effectively assesses cerebral blood flow velocity and abnormalities of the venous lumen. TCD recognizes venous blood vessels by relying on fragments of the circle of Willis as markers, and thus is limited in the scope of examinations it permits. The advantage of TCCS over TCD is that it can depict venous structures using blood flow information independently of the need for an arterial marker. In addition, TCCS may be used to study the collateral circulation of intracranial veins. Given its inexpensiveness and non-invasiveness, TCCS can also be used as a detection method in follow-up patients. However, TCCS does have some drawbacks. First, it cannot detect all intracranial venous structures: for older patients, thickening of the bony structures affects the reflection of sound waves, resulting in interference that impedes detection of intracranial veins. In addition, increased intracranial pressure affects hemodynamics, thus affecting detection with TCCS. Finally, there are a large number of variations in the intracranial veins, especially among smaller vessels, that are not supported by uniform norm data and therefore difficult to evaluate with TCCS [125-128].

#### 2.3.4 Four-dimensional flow magnetic resonance imaging (4D-MRI)

Despite its advantages, one drawback of MRV is its lack of capacity to collect quantitative hemodynamic information; 4-dimensional (4D) flow MRI, by contrast, offers the potential to visualize and quantify the hemodynamics of the entire cerebral venous system. 4D-MRI provides unique and detailed insights, including changes in hemodynamics, recruitment of collateral pathways, and normalization of blood flow conditions after recanalization. Thus, this technique overcomes the limitations of standard MRV and CTV with respect to detecting hemodynamic information, as well as the limitations of ultrasound regarding bony windows that may hinder the detection of intracranial veins [78, 129-132].

#### 2.3.5 Four-dimensional CT angiography (4D-CTA)

4D-CTA is a convenient non-invasive alternative to DSA that can both identify cerebral venous abnormalities such as CVT or CVS stenosis, and is sufficiently sensitive to differentiate between them. In comparison with angiographic information obtained with DSA and MRV, however, its ability to image subependymal and deep medullary veins is suboptimal [133].

#### 2.3.6 Intravascular ultrasound (IVUS)

IVUS is an effective diagnostic tool in coronary and carotid angiography as well as in endovascular stenting that has also been applied in recent years to cerebral venous diseases. As an auxiliary tool for conventional venoplasty or stenting, IVUS appears to possess potential to augment diagnostic accuracy and to provide management guidance [134, 135].

### 2.4 Treatment

Currently, endovascular stenting is the most widely applied treatment for CVS stenosis. Although stenting has been shown to effectively alleviate CVS stenosis-related symptoms such as intracranial hypertension and tinnitus [6-10, 18-20, 136], assessment of its overall safety and efficacy will require larger, multicenter clinical trials. As more patients with CVS stenosis undergo this procedure, specification of the ideal candidate profile for stenting, as well as a comprehensive account of its risks, benefits, and potential complications, will serve as objectives for future research.

As for intracranial mass lesions such as meningiomas, optimization of surgical strategy may improve clinical results and minimize complications such as the need for sinus reconstruction [102-105]. One apparently promising surgical technique for patients with CVS stenosis is cerebrospinal fluid shunt placement. Levitt et al. reported that a patient with intracranial hypertension had a significant improvement in bilateral transverse sinus stenosis after installation of a cerebrospinal fluid shunt, leading the authors to speculate that intracranial hypertension may cause collapse of the transverse sinus wall, resulting in stenosis. Reduction of intracranial pressure using a cerebrospinal fluid shunt may reduce the pressure gradient to which the venous wall is exposed, allowing the sinus to re-expand [137].

## 3. Internal jugular vein (IJV) stenosis

### 3.1 Proposed etiology

Unlike the internal carotid artery, the internal jugular vein lacks smooth muscle, rendering it vulnerable to extrinsic impingement. Thus, as was the case for CVS stenosis, the etiology of IJV stenosis can be divided into extrinsic and intrinsic anomalies.

#### 3.1.1 Extrinsic anomalies

Structural abnormalities adjacent to the IJV may manifest as bony compression, arterial compression, enlarged lymph nodes, or an enlarged thyroid, and may therefore impinge on the venous wall, causing venous outflow obstruction [123, 138]. Compression of the IJV lumen may produce a spectrum of consequences ranging from local stenosis to complete occlusion. The etiology of compressive insults to the IJV varies along its different segments. In general, the upper IJV is susceptible to compression by the lateral mass of cervical vertebrae at the C1 segment and by the styloid processes, while the middle and lower IJV are more susceptible to compression by the adjacent carotid artery, lymph nodes, and aberrant muscles. Of all these potentially impinging structures, bone appears to be the most common culprit, with previous studies showing that about 40% of extrinsic anomalies are bony in origin [14, 15]. A recent study showed that the incidence of IJV stenosis secondary to external compression reached 41.9% in a Chinese cohort [139].

#### 3.1.2 Intrinsic anomalies

Intrinsic anomalies include thrombi, vessel wall abnormalities, and malformed venous valves. IJV thrombosis is a rare entity, mentioned only in a few case reports [140-142]. Intraluminal defects (such as flaps, webs, septa, membranes, and malformed valves) may hinder normal blood flow drainage from the brain, resulting in hemodynamic alterations, including reflux, reduced flow, or no flow. Doppler and intravascular ultrasound are effective tools for visualizing malformed valves, of which a variety of types have been reported, including fused, elongated, ectopic, accessory leaflet-containing, inverted, and double valves [143, 144].

#### 3.1.3 The vertebral venous system (VVS)

The VVS is a valveless, interconnected venous system with abundant side branch anastomoses that extends longitudinally down the entire length of the spinal canal. It can be partitioned into three distinct divisions: an internal plexus, an external plexus, and the basivertebral veins. Postural changes between the supine and erect positions affect the drainage of the VVS. In the erect position, venous outflow from the brain mainly relies on VVS, and the IJVs only drain approximately 10% of outflow. Thus, the VVS provides free venous communication between the brain and spinal cord, and could potentially serve as an alternative circulatory route in patient with IJV stenosis. However, it also increases the risk of tumor metastasis, infection and embolism [145-147].

#### 4.1.4 IJV stenosis and CNS disorders

It has been reported that IJV stenosis may be associated with various CNS disorders, such as multiple sclerosis, Alzheimer's disease, Parkinson's disease, and Meniere's disease [148-152]. In the case of MS, the link with IJV stenosis has, since it was proposed by Zamboni P et al. in 2009 along with the concept of chronic cerebrospinal venous insufficiency (CCVSI), triggered a wide-ranging debate. One of the outcomes of his conception, according to which abnormalities in cerebrospinal venous flow may contribute to the pathogenesis of MS, was that an increasing number of MS patients undergo endovascular treatment in an attempt to improve neurological function and quality of life. Unfortunately, recent multicenter randomized controlled trials have concluded that endovascular treatment does not improve the outcome of MS, and suggested that endovascular treatment for MS patients should be discouraged until appropriate high-quality trials confirm safety and efficacy [153]. Another perspective on CCVSI was provided by Thibault et al., who proposed that chronic persistent venulitis caused by Chlamydophila pneumoniae, an obligate intracellular bacterium, may play a role in CCVSI and other vascular diseases; their non-randomized cohort study found that extracranial blood flow improved after treatment with a combined antibiotic protocol [154-156]. In summary, although some CNS disorders appear to be associated with IJV stenosis, causal relationships between these entities and IJV stenosis have not been firmly established. To further evaluate this issue, comprehensive consideration of precipitating risks such as age, sex, race, comorbidities, etc., should be incorporated into future safe and ethical observational studies.

### 3.2 Clinical manifestations

The clinical manifestations of IJV outflow disturbance may differ among patients due to anatomical variation and variable compensatory capacity of collaterals. Clinical manifestations such as headache, head noises, tinnitus, visual disorders, hearing loss, sleep disturbances, cognitive decline, and neck discomfort are common in IJV stenosis. Of these, head noises and tinnitus may be the typical clinical manifestations of IJV stenosis and are suggestive of IJV blood flow alterations. IJV stenosis can also precipitate intracranial hypertension, which can be effectively relieved by stenting [14, 15, 19].

**Figure 4.**
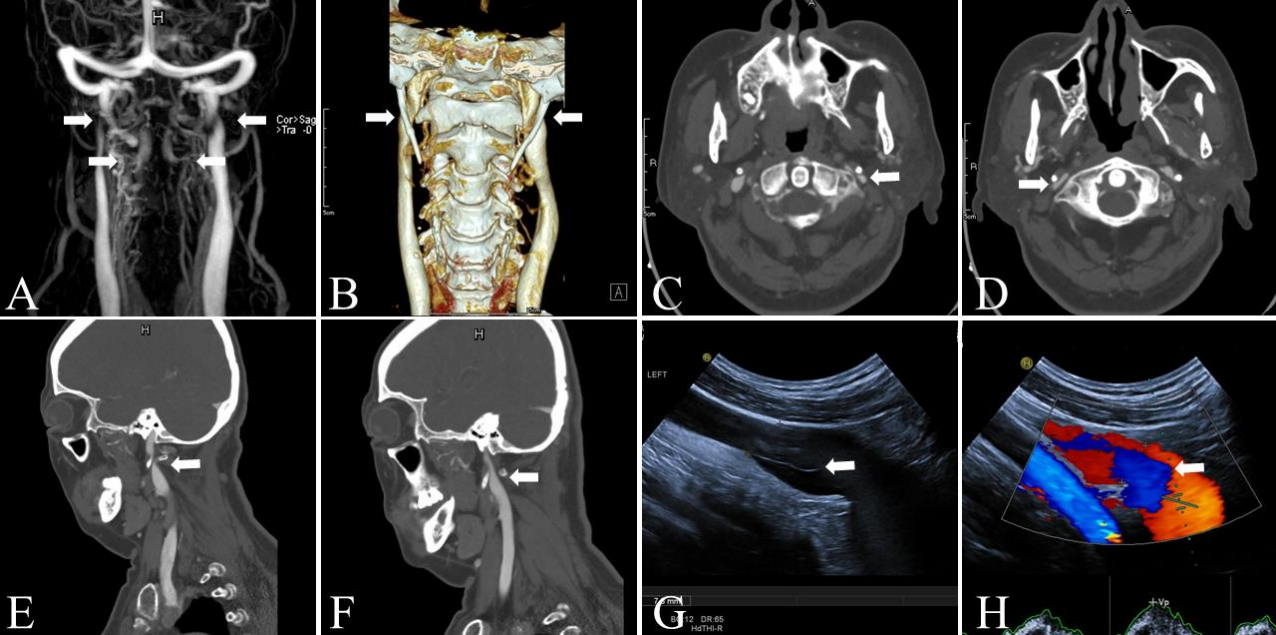
Imaging findings in patients with IJV stenosis. (A-F) Bony compression-induced bilateral IJV stenosis seen with MRV and CTV. Bilateral internal jugular vein stenosis with abnormally tortuous vertebral venous plexus compensation seen with MRV (A). CTV showed that the bilateral internal jugular veins were significantly compressed by elongated styloid processes, as well as the transverse processes of C1 (B-F). (G-H) Lengthy internal jugular vein valve that induced IJV reflux. Jugular venous ultrasound showed a lengthy venous valve (G) and the blood reflux it precipitated (H).

### 3.3 Diagnosis

#### 3.3.1 MRV

MRV is the most commonly used technique for assessing extracranial vessels and provides the advantage of non-invasiveness as well as of less operator dependence. TOF-MRV can be performed quickly to directly visualize IJV morphology and is more sensitive in the presence of slow blood flow. CE-MRV, however can visualize IJV anatomy in more detail. In addition, 4D-MRI and Phase-Contrast (PC)-MRV have the potential to assess IJV blood flow velocity and pattern [157-159].

#### 3.3.2 Doppler ultrasonography

Doppler ultrasonography is the primary screening tool used in the evaluation for IJV stenosis. Its benefits include its non-invasiveness, low cost, and freedom from radiation; it is also useful for providing information on IJV flow velocity and assessing for the presence of any abnormal intravascular structures. Its use is hindered, however, by operator dependence, which may result in variable measurements [160, 161]. Another potential application for this technology was proposed by Thibault et al., who described the efficacy of extracranial duplex ultrasonography for the postoperative assessment of venoplasty. They found that ultrasonography provided reliable information about IJV and vertebral vein stenoses and could, with the help of venous blood volume flow detection technology, quantify the degree of stenosis [162].

Ultrasound may also be used in tandem with venography in a technique referred to as intravascular ultrasound (IVUS). Compared with conventional venography, IVUS provided higher resolution and measurement accuracy to better distinguish true stenotic lesions, while venography yielded some false positives. In addition, IVUS was found to be superior to conventional venography in displaying intravascular abnormalities, resulting in higher diagnostic accuracy [163, 164] (Fig. 4).

#### 3.3.3 Catheter venography

Catheter venography is still considered the gold standard for assessment of the degree of IJV stenosis and pressure gradients in patients scheduled for endovascular surgery. However, as discussed above, it is inferior to IVUS in reflecting intravascular anomalies. Moreover, the contrast injection and exposure to radiation it requires limit the desirability of catheter venography as a method for evaluating IJV stenosis [163-167].

### 3.4 Treatment

To date, a deficiency of strong clinical evidence has impaired the production of a consensus with respect to the treatment of IJV stenosis. Current treatments mainly attempt to address etiologies and symptoms, with the aim of restoring normal IJV hemodynamics.

#### 3.4.1 IJV thrombosis

Although IJV thrombosis is not common, timely anticoagulation or endovascular treatment appears, in the interest of avoiding serious complications, to be advisable. Standard anticoagulant therapy, consisting of subcutaneous injections of low molecular weight heparin or intravenous heparin for several days with a subsequent transition to oral anticoagulants, is recommended [141, 142, 168, 169]. It should be understood that these treatment recommendations are not limited to simple IJV thrombosis: other applicable situations include planning IJV reconstruction surgery, IJV venous valve malformations and other hemodynamic abnormalities related to IJV stenosis that predispose to thrombosis and coexisting hypercoagulable states.

#### 3.4.2 Extrinsic abnormalities

Extrinsic impingement-mediated IJV stenosis may require a different treatment approach. Given the large proportion of such cases that appear to be caused by bony compression, it is important to evaluate for this etiology in patients with non-thrombotic IJV stenosis. Multidisciplinary cooperation is gradually emerging as important in this context in the interest of successfully restoring IJV hemodynamics. For example, in cases caused by bony compression from the styloid process and the lateral mass of the C1 cervical vertebral segment, endovascular stenting alone appears to be ineffective secondary to an inability to expand. Thus, modified styloidectomy is considered as a potential adjunct approach to alleviate the compression. In addition, external compression of the IJV by aberrant muscular elements has also been reported, and may similarly require adjunctive approaches in addition to stenting to avoid frustrated expansion [170, 171].

#### 3.4.3 Intraluminal defects

Intraluminal defects leading to impaired IJV blood flow may be managed with percutaneous angioplasty techniques such as endophlebectomy and autologous vein patch angioplasty, which appear to alleviate blood flow problems within the IJV,[172, 173] but are currently in need of stronger clinical evidence to support their safety and efficacy.

#### 3.4.4 Endovascular treatment

Zamboni P et al. reported in 2009 that chronic cerebrospinal venous insufficiency might, as mentioned above, be responsible for multiple sclerosis [148], but the endovascular stenting and balloon angioplasty that have since emerged as therapies are controversial [173-176]. Large, multicenter randomized controlled trials have concluded that endovascular treatment might be safe but ineffective for patients with multiple sclerosis [177-179]; moreover, significant adverse events and complications of stenting performed for this purpose have been recorded [180]. On the other hand, one study performed on a Chinese cohort revealed the encouraging result that IJV stenosis-induced intracranial hypertension can be corrected by stenting [19]. Therefore, the development of more rigorous inclusion criteria and careful assessment of potential complications for patients with IJV stenosis who may benefit from stenting is urgently needed.

#### 3.4.5 Other treatments

Because of sufficient collateral circulation, a considerable number of patients with IJV stenosis have normal or only mildly elevated intracranial pressure [15]. By contrast, some patients with IJV stenosis may present with severe intracranial hypertension resulting in serious visual damage. For these patients, optic nerve sheath fenestration may be an effective method of alleviating risk of visual deterioration in cases when the causative IJV stenosis cannot be corrected immediately [61, 181].

## 4. Summary

In conclusion, intra- and/or extra-cranial venous outflow disturbances merit more scientific attention than they have received up to this point. This is due, in addition to the intrinsic desirability of further characterizing these important categories of cerebrovascular pathology, to the avoidance of misdiagnosis and treatment delay that a comprehensive understanding of these entities could help achieve. Currently, multicenter, case-control studies designed to assess treatment of cerebrovenous outflow disorders are limited, and research regarding etiologies and pathophysiological mechanisms is, similarly, far from sufficient. It is therefore essential that large, multi-center, well-designed randomized and controlled clinical trials be designed as soon as possible to rectify these gaps in understanding, as they may make a substantial difference to the treatment of our future patients.

## References

[1] Ferro JM, Canhao P, Stam J, Bousser MG, Barinagarrementeria F (2004). Prognosis of cerebral vein and dural sinus thrombosis: results of the International Study on Cerebral Vein and Dural Sinus Thrombosis (ISCVT). Stroke, 35:664-670. 14976332 10.1161/01.STR.0000117571.76197.26

[2] Luo Y, Tian X, Wang X (2018). Diagnosis and Treatment of Cerebral Venous Thrombosis: A Review. Front Aging Neurosci, 10:2. 29441008 10.3389/fnagi.2018.00002 PMC5797620

[3] Mahale R, Mehta A, John AA, Buddaraju K, Shankar AK, Javali M, et al (2016). Acute seizures in cerebral venous sinus thrombosis: What predicts it? Epilepsy Res, 123:1-5. 27023399 10.1016/j.eplepsyres.2016.01.011

[4] Long B, Koyfman A, Runyon MS (2017). Cerebral Venous Thrombosis: A Challenging Neurologic Diagnosis. Emerg Med Clin North Am, 35:869-878. 28987433 10.1016/j.emc.2017.07.004

[5] Kowoll CM, Kaminski J, Weiss V, Bosel J, Dietrich W, Juttler E, et al (2016). Severe Cerebral Venous and Sinus Thrombosis: Clinical Course, Imaging Correlates, and Prognosis. Neurocrit Care, 25:392-399. 27000641 10.1007/s12028-016-0256-8

[6] Cappuzzo JM, Hess RM, Morrison JF, Davies JM, Snyder KV, Levy EI, et al (2018). Transverse venous stenting for the treatment of idiopathic intracranial hypertension, or pseudotumor cerebri. Neurosurg Focus, 45:E11. 10.3171/2018.5.FOCUS18102 29961386

[7] Higgins JN, Cousins C, Owler BK, Sarkies N, Pickard JD (2003). Idiopathic intracranial hypertension: 12 cases treated by venous sinus stenting. J Neurol Neurosurg Psychiatry, 74:1662-1666. 14638886 10.1136/jnnp.74.12.1662 PMC1757418

[8] Fargen KM, Liu K, Garner RM, Greeneway GP, Wolfe SQ, Crowley RW (2018). Recommendations for the selection and treatment of patients with idiopathic intracranial hypertension for venous sinus stenting. J Neurointerv Surg, 10:1203-1208. 30030306 10.1136/neurintsurg-2018-014042

[9] Puffer RC, Mustafa W, Lanzino G (2013). Venous sinus stenting for idiopathic intracranial hypertension: a review of the literature. J Neurointerv Surg, 5:483-486. 22863980 10.1136/neurintsurg-2012-010468

[10] Raper DMS, Buell TJ, Ding D, Pomeraniec IJ, Crowley RW, Liu KC (2018). A pilot study and novel angiographic classification for superior sagittal sinus stenting in patients with non-thrombotic intracranial venous occlusive disease. J Neurointerv Surg, 10:74-77. 28082447 10.1136/neurintsurg-2016-012906

[11] Farb RI, Vanek I, Scott JN, Mikulis DJ, Willinsky RA, Tomlinson G, et al (2003). Idiopathic intracranial hypertension: the prevalence and morphology of sinovenous stenosis. Neurology, 60:1418-1424. 12743224 10.1212/01.wnl.0000066683.34093.e2

[12] Giridharan N, Patel SK, Ojugbeli A, Nouri A, Shirani P, Grossman AW, et al (2018). Understanding the complex pathophysiology of idiopathic intracranial hypertension and the evolving role of venous sinus stenting: a comprehensive review of the literature. Neurosurg Focus, 45:E10. 10.3171/2018.4.FOCUS18100 29961379

[13] West JL, Greeneway GP, Garner RM, Aschenbrenner CA, Singh J, Wolfe SQ, et al (2019). Correlation between angiographic stenosis and physiologic venous sinus outflow obstruction in idiopathic intracranial hypertension. J Neurointerv Surg, 11:90-94. 29858399 10.1136/neurintsurg-2018-014004

[14] Zhou D, Ding J, Asmaro K, Pan L, Ya J, Yang Q, et al (2019). Clinical Characteristics and Neuroimaging Findings in Internal Jugular Venous Outflow Disturbance. Thromb Haemost, 119:308-318. 30605919 10.1055/s-0038-1676815

[15] Bai C, Xu Y, Zhou D, Ding J, Yang Q, Ding Y, et al (2019). The comparative analysis of non-thrombotic internal jugular vein stenosis and cerebral venous sinus stenosis. J Thromb Thrombolysis. 10.1007/s11239-019-01820-1 30689154

[16] Lenck S, Vallee F, Civelli V, Saint-Maurice JP, Nicholson P, Hong A, et al (2018). Assessment of blood flow velocities and venous pressures using a dual-sensor guidewire in symptomatic dural sinus stenoses. J Neurosurg: 1-5. 10.3171/2017.12.JNS172364 29999456

[17] Kefayati S, Amans M, Faraji F, Ballweber M, Kao E, Ahn S, et al (2017). The manifestation of vortical and secondary flow in the cerebral venous outflow tract: An in vivo MR velocimetry study. J Biomech, 50:180-187. 27894675 10.1016/j.jbiomech.2016.11.041 PMC5191981

[18] Baomin L, Yongbing S, Xiangyu C (2014). Angioplasty and stenting for intractable pulsatile tinnitus caused by dural venous sinus stenosis: a case series report. Otol Neurotol, 35:366-370. 24080976 10.1097/MAO.0b013e3182990d52

[19] Zhou D, Meng R, Zhang X, Guo L, Li S, Wu W, et al (2018). Intracranial hypertension induced by internal jugular vein stenosis can be resolved by stenting. Eur J Neurol, 25:365-e313. 29114973 10.1111/ene.13512

[20] Xu K, Yu T, Yuan Y, Yu J (2015). Current Status of the Application of Intracranial Venous Sinus Stenting. Int J Med Sci, 12:780-789. 26516306 10.7150/ijms.12604 PMC4615238

[21] Silvis SM, Lindgren E, Hiltunen S, Devasagayam S, Scheres LJ, Jood K, et al (2019). Postpartum Period Is a Risk Factor for Cerebral Venous Thrombosis. Stroke, 50:501-503. 30621526 10.1161/STROKEAHA.118.023017

[22] Duman T, Uluduz D, Midi I, Bektas H, Kablan Y, Goksel BK, et al (2017). A Multicenter Study of 1144 Patients with Cerebral Venous Thrombosis: The VENOST Study. J Stroke Cerebrovasc Dis, 26:1848-1857. 28583818 10.1016/j.jstrokecerebrovasdis.2017.04.020

[23] Pan L, Ding J, Ya J, Zhou D, Hu Y, Fan C, et al (2019). Risk factors and predictors of outcomes in 243 Chinese patients with cerebral venous sinus thrombosis: A retrospective analysis. Clin Neurol Neurosurg, 183:105384. 31229936 10.1016/j.clineuro.2019.105384

[24] Dentali F, Crowther M, Ageno W (2006). Thrombophilic abnormalities, oral contraceptives, and risk of cerebral vein thrombosis: a meta-analysis. Blood, 107:2766-2773. 16397131 10.1182/blood-2005-09-3578

[25] Amoozegar F, Ronksley PE, Sauve R, Menon BK (2015). Hormonal contraceptives and cerebral venous thrombosis risk: a systematic review and meta-analysis. Front Neurol, 6:7. 25699010 10.3389/fneur.2015.00007 PMC4313700

[26] Silvis SM, Hiltunen S, Lindgren E, Jood K, Zuurbier SM, Middeldorp S, et al (2018). Cancer and risk of cerebral venous thrombosis: a case-control study. J Thromb Haemost, 16:90-95. 29125690 10.1111/jth.13903

[27] Zuurbier SM, Arnold M, Middeldorp S, Broeg-Morvay A, Silvis SM, Heldner MR, et al (2016). Risk of Cerebral Venous Thrombosis in Obese Women. JAMA Neurol, 73:579-584. 26974867 10.1001/jamaneurol.2016.0001

[28] Silvis SM, de Sousa DA, Ferro JM, Coutinho JM (2017). Cerebral venous thrombosis. Nat Rev Neurol, 13:555-565. 28820187 10.1038/nrneurol.2017.104

[29] Silvis SM, Middeldorp S, Zuurbier SM, Cannegieter SC, Coutinho JM (2016). Risk Factors for Cerebral Venous Thrombosis. Semin Thromb Hemost, 42:622-631. 27272966 10.1055/s-0036-1584132

[30] Lauw MN, Barco S, Coutinho JM, Middeldorp S (2013). Cerebral venous thrombosis and thrombophilia: a systematic review and meta-analysis. Semin Thromb Hemost, 39:913-927. 24129682 10.1055/s-0033-1357504

[31] Martinelli I, Mannucci PM, De Stefano V, Taioli E, Rossi V, Crosti F, et al (1998). Different risks of thrombosis in four coagulation defects associated with inherited thrombophilia: a study of 150 families. Blood, 92:2353-2358. 9746774

[32] Aznar J, Vaya A, Estelles A, Mira Y, Segui R, Villa P, et al (2000). Risk of venous thrombosis in carriers of the prothrombin G20210A variant and factor V Leiden and their interaction with oral contraceptives. Haematologica, 85:1271-1276. 11114134

[33] Green M, Styles T, Russell T, Sada C, Jallow E, Stewart J, et al (2018). Non-genetic and genetic risk factors for adult cerebral venous thrombosis. Thromb Res, 169:15-22. 30005273 10.1016/j.thromres.2018.07.005

[34] Vecht L, Zuurbier SM, Meijers JCM, Coutinho JM (2018). Elevated factor VIII increases the risk of cerebral venous thrombosis: a case-control study. J Neurol, 265:1612-1617. 29737426 10.1007/s00415-018-8887-7

[35] Reuner KH, Jenetzky E, Aleu A, Litfin F, Mellado P, Kloss M, et al (2008). Factor XII C46T gene polymorphism and the risk of cerebral venous thrombosis. Neurology, 70:129-132. 18180442 10.1212/01.wnl.0000296825.05176.da

[36] Prabhakar P, De T, Nagaraja D, Christopher R (2012). Association of factor XII gene C46T polymorphism with cerebral venous thrombosis in the south Indian population. J Thromb Haemost, 10:1437-1439. 22500857 10.1111/j.1538-7836.2012.04743.x

[37] Bellucci S, Cassinat B, Bonnin N, Marzac C, Crassard I (2008). The V617F JAK 2 mutation is not a frequent event in patients with cerebral venous thrombosis without overt chronic myeloproliferative disorder. Thromb Haemost, 99:1119-1120. 18521518 10.1160/TH08-02-0081

[38] De Stefano V, Rossi E, Za T, Chiusolo P, Leone G (2008). The JAK2 V617F mutation in patients with cerebral venous thrombosis: a rebuttal. Thromb Haemost, 99:1121. 18521519 10.1160/TH08-04-0205

[39] Passamonti SM, Biguzzi E, Cazzola M, Franchi F, Gianniello F, Bucciarelli P, et al (2012). The JAK2 V617F mutation in patients with cerebral venous thrombosis. J Thromb Haemost, 10:998-1003. 22469236 10.1111/j.1538-7836.2012.04719.x

[40] Liu XY, Gabig TG, Bang NU (2000). Combined heterozygosity of factor V leiden and the G20210A prothrombin gene mutation in a patient with cerebral cortical vein thrombosis. Am J Hematol, 64:226-228. 10861823 10.1002/1096-8652(200007)64:3<226::aid-ajh17>3.0.co;2-f

[41] Sahu KK, Varma SC (2015). Cortical vein thrombosis in a case of idiopathic thrombocytopenic purpura. Platelets, 26:374-375. 24678621 10.3109/09537104.2014.898180

[42] Chang R, Friedman DP (2004). Isolated cortical venous thrombosis presenting as subarachnoid hemorrhage: a report of three cases. AJNR Am J Neuroradiol, 25:1676-1679. 15569729 PMC8148749

[43] Sagduyu A, Sirin H, Mulayim S, Bademkiran F, Yunten N, Kitis O, et al (2006). Cerebral cortical and deep venous thrombosis without sinus thrombosis: clinical MRI correlates. Acta Neurol Scand, 114:254-260. 16942545 10.1111/j.1600-0404.2006.00595.x

[44] Bushnell C, Saposnik G (2014). Evaluation and management of cerebral venous thrombosis. Continuum (Minneap Minn), 20:335-351. 24699485 10.1212/01.CON.0000446105.67173.a8 PMC10564072

[45] Murray BJ, Llinas R, Caplan LR, Scammell T, Pascual-Leone A (2000). Cerebral deep venous thrombosis presenting as acute micrographia and hypophonia. Neurology, 54:751-753. 10680819 10.1212/wnl.54.3.751

[46] Rafique MZ, Bari V, Ashraf K, Ahmad MN (2005). Cerebral deep venous thrombosis: case report and literature review. J Pak Med Assoc, 55:399-400. 16302476

[47] Sparaco M, Feleppa M, Bigal ME (2015). Cerebral Venous Thrombosis and Headache--A Case-Series. Headache, 55:806-814. 26084237 10.1111/head.12599

[48] Kumral E, Polat F, Uzunkopru C, Calli C, Kitis O (2012). The clinical spectrum of intracerebral hematoma, hemorrhagic infarct, non-hemorrhagic infarct, and non-lesional venous stroke in patients with cerebral sinus-venous thrombosis. Eur J Neurol, 19:537-543. 22035069 10.1111/j.1468-1331.2011.03562.x

[49] Coutinho JM, Stam J, Canhao P, Barinagarrementeria F, Bousser MG, Ferro JM (2015). Cerebral venous thrombosis in the absence of headache. Stroke, 46:245-247. 25378420 10.1161/STROKEAHA.114.007584

[50] Wasay M, Kojan S, Dai AI, Bobustuc G, Sheikh Z (2010). Headache in Cerebral Venous Thrombosis: incidence, pattern and location in 200 consecutive patients. J Headache Pain, 11:137-139. 20112042 10.1007/s10194-010-0186-3 PMC3452295

[51] Timoteo A, Inacio N, Machado S, Pinto AA, Parreira E (2012). Headache as the sole presentation of cerebral venous thrombosis: a prospective study. J Headache Pain, 13:487-490. 22592865 10.1007/s10194-012-0456-3 PMC3464467

[52] Costa P, Del Zotto E, Giossi A, Volonghi I, Poli L, Frigerio M, et al (2012). Headache due to spontaneous intracranial hypotension and subsequent cerebral vein thrombosis. Headache, 52:1592-1596. 23046074 10.1111/j.1526-4610.2012.02261.x

[53] Saposnik G, Barinagarrementeria F, Brown RD Jr, Bushnell CD, Cucchiara B, Cushman M, et al (2011). Diagnosis and management of cerebral venous thrombosis: a statement for healthcare professionals from the American Heart Association/American Stroke Association. Stroke, 42:1158-1192. 21293023 10.1161/STR.0b013e31820a8364

[54] Ferro JM, Correia M, Rosas MJ, Pinto AN, Neves G (2003). Seizures in cerebral vein and dural sinus thrombosis. Cerebrovasc Dis, 15:78-83. 12499715 10.1159/000067133

[55] Masuhr F, Busch M, Amberger N, Ortwein H, Weih M, Neumann K, et al (2006). Risk and predictors of early epileptic seizures in acute cerebral venous and sinus thrombosis. Eur J Neurol, 13:852-856. 16879295 10.1111/j.1468-1331.2006.01371.x

[56] Sha DJ, Qian J, Gu SS, Wang LN, Wang F, Xu Y (2018). Cerebral venous sinus thrombosis complicated by seizures: a retrospective analysis of 69 cases. J Thromb Thrombolysis, 45:186-191. 29039017 10.1007/s11239-017-1570-5 PMC5756278

[57] Coutinho JM, Zuurbier SM, Stam J (2014). Declining mortality in cerebral venous thrombosis: a systematic review. Stroke, 45:1338-1341. 24699058 10.1161/STROKEAHA.113.004666

[58] Rashad S, Niizuma K, Sato-Maeda M, Fujimura M, Mansour A, Endo H, et al (2018). Early BBB breakdown and subacute inflammasome activation and pyroptosis as a result of cerebral venous thrombosis. Brain Res. 10.1016/j.brainres.2018.06.029 29981290

[59] Pfefferkorn T, Crassard I, Linn J, Dichgans M, Boukobza M, Bousser MG (2009). Clinical features, course and outcome in deep cerebral venous system thrombosis: an analysis of 32 cases. J Neurol, 256:1839-1845. 19536581 10.1007/s00415-009-5206-3

[60] Wasay M, Bakshi R, Bobustuc G, Kojan S, Sheikh Z, Dai A, et al (2008). Cerebral venous thrombosis: analysis of a multicenter cohort from the United States. J Stroke Cerebrovasc Dis, 17:49-54. 18346644 10.1016/j.jstrokecerebrovasdis.2007.10.001

[61] Peng KP, Fuh JL, Wang SJ (2012). High-pressure headaches: idiopathic intracranial hypertension and its mimics. Nat Rev Neurol, 8:700-710. 23165338 10.1038/nrneurol.2012.223

[62] Thammishetti V, Dharanipragada S, Basu D, Ananthakrishnan R, Surendiran D (2016). A Prospective Study of the Clinical Profile, Outcome and Evaluation of D-dimer in Cerebral Venous Thrombosis. J Clin Diagn Res, 10:Oc07-10. 10.7860/JCDR/2016/19114.7926 PMC4963685 27504325

[63] Hassan KM, Kumar D (2013). Reversible diencephalic dysfunction as presentation of deep cerebral venous thrombosis due to hyperhomocysteinemia and protein S deficiency: Documentation of a case. J Neurosci Rural Pract, 4:193-196. 23914104 10.4103/0976-3147.112767 PMC3724306

[64] Sharma RC, Kainth A, Sharma S (2015). Transient Global Amnesia as a Presenting Manifestation of Cerebral Venous Thrombosis. J Neuropsychiatry Clin Neurosci, 27:e209-210. 26222971 10.1176/appi.neuropsych.14110354

[65] Ford K, Sarwar M (1981). Computed tomography of dural sinus thrombosis. AJNR Am J Neuroradiol, 2:539-543. 6797278 PMC8335245

[66] Linn J, Ertl-Wagner B, Seelos KC, Strupp M, Reiser M, Bruckmann H, et al (2007). Diagnostic value of multidetector-row CT angiography in the evaluation of thrombosis of the cerebral venous sinuses. AJNR Am J Neuroradiol, 28:946-952. 17494676 PMC8134361

[67] Khandelwal N, Agarwal A, Kochhar R, Bapuraj JR, Singh P, Prabhakar S, et al (2006). Comparison of CT venography with MR venography in cerebral sinovenous thrombosis. AJR Am J Roentgenol, 187:1637-1643. 17114562 10.2214/AJR.05.1249

[68] Majoie CB, van Straten M, Venema HW, den Heeten GJ (2004). Multisection CT venography of the dural sinuses and cerebral veins by using matched mask bone elimination. AJNR Am J Neuroradiol, 25:787-791. 15140721 PMC7974470

[69] Lafitte F, Boukobza M, Guichard JP, Hoeffel C, Reizine D, Ille O, et al (1997). MRI and MRA for diagnosis and follow-up of cerebral venous thrombosis (CVT). Clin Radiol, 52:672-679. 9313731 10.1016/s0009-9260(97)80030-x

[70] Selim M, Fink J, Linfante I, Kumar S, Schlaug G, Caplan LR (2002). Diagnosis of cerebral venous thrombosis with echo-planar T2*-weighted magnetic resonance imaging. Arch Neurol, 59:1021-1026. 12056941 10.1001/archneur.59.6.1021

[71] Klingebiel R, Bauknecht HC, Bohner G, Kirsch R, Berger J, Masuhr F (2007). Comparative evaluation of 2D time-of-flight and 3D elliptic centric contrast-enhanced MR venography in patients with presumptive cerebral venous and sinus thrombosis. Eur J Neurol, 14:139-143. 17250720 10.1111/j.1468-1331.2006.01574.x

[72] Paoletti M, Germani G, De Icco R, Asteggiano C, Zamboni P, Bastianello S (2016). Intra- and Extracranial MR Venography: Technical Notes, Clinical Application, and Imaging Development. Behav Neurol, 2016:2694504. 27340338 10.1155/2016/2694504 PMC4906191

[73] Salehi Ravesh M, Jensen-Kondering U, Juhasz J, Peters S, Huhndorf M, Graessner J, et al (2019). Optimization of 3D phase contrast venography for the assessment of the cranio-cervical venous system at 1.5 T. Neuroradiology, 61:293-304. 30607475 10.1007/s00234-018-2146-6

[74] Tsai FY, Kostanian V, Rivera M, Lee KW, Chen CC, Nguyen TH (2007). Cerebral venous congestion as indication for thrombolytic treatment. Cardiovasc Intervent Radiol, 30:675-687. 17573553 10.1007/s00270-007-9046-1

[75] Tsai FY, Nguyen B, Lin WC, Hsueh CJ, Yen A, Meng K, et al (2008). Endovascular procedures for cerebrovenous disorders. Acta Neurochir Suppl, 101:83-86. 18642639 10.1007/978-3-211-78205-7_14

[76] Hsu HY, Wang PY, Chen CC, Hu HH (2004). Dural arteriovenous fistula after cerebral sinus thrombosis: a case study of serial venous transcranial color-coded sonography. J Ultrasound Med, 23:1095-1100. 15284469 10.7863/jum.2004.23.8.1095

[77] Komiyama M, Ishiguro T, Kitano S, Sakamoto H, Nakamura H (2004). Serial antenatal sonographic observation of cerebral dural sinus malformation. AJNR Am J Neuroradiol, 25:1446-1448. 15466350 PMC7975451

[78] Schuchardt F, Schroeder L, Anastasopoulos C, Markl M, Bauerle J, Hennemuth A, et al (2015). In vivo analysis of physiological 3D blood flow of cerebral veins. Eur Radiol, 25:2371-2380. 25638218 10.1007/s00330-014-3587-x

[79] Boukobza M, Crassard I, Bousser MG, Chabriat H (2009). MR imaging features of isolated cortical vein thrombosis: diagnosis and follow-up. AJNR Am J Neuroradiol, 30:344-348. 19095790 10.3174/ajnr.A1332 PMC7051397

[80] Yang Q, Duan J, Fan Z, Qu X, Xie Y, Nguyen C, et al (2016). Early Detection and Quantification of Cerebral Venous Thrombosis by Magnetic Resonance Black-Blood Thrombus Imaging. Stroke, 47:404-409. 26670082 10.1161/STROKEAHA.115.011369 PMC4729606

[81] Niu PP, Yu Y, Guo ZN, Jin H, Liu Y, Zhou HW, et al (2016). Diagnosis of non-acute cerebral venous thrombosis with 3D T1-weighted black blood sequence at 3T. J Neurol Sci, 367:46-50. 27423563 10.1016/j.jns.2016.05.052

[82] Vatankhah B, Furst A, Schlachetzki F (2005). Do normal d-dimer levels reliably exclude cerebral sinus thrombosis? A solution of problems? Stroke, 36:2528-2529; author reply 2529. 16304137

[83] Lalive PH, de Moerloose P, Lovblad K, Sarasin FP, Mermillod B, Sztajzel R (2003). Is measurement of D-dimer useful in the diagnosis of cerebral venous thrombosis? Neurology, 61:1057-1060. 14581664 10.1212/01.wnl.0000090562.66120.1f

[84] Dentali F, Squizzato A, Marchesi C, Bonzini M, Ferro JM, Ageno W (2012). D-dimer testing in the diagnosis of cerebral vein thrombosis: a systematic review and a meta-analysis of the literature. J Thromb Haemost, 10:582-589. 22257124 10.1111/j.1538-7836.2012.04637.x

[85] Crassard I, Soria C, Tzourio C, Woimant F, Drouet L, Ducros A, et al (2005). A negative D-dimer assay does not rule out cerebral venous thrombosis: a series of seventy-three patients. Stroke, 36:1716-1719. 16020765 10.1161/01.STR.0000173401.76085.98

[86] Alons IM, Jellema K, Wermer MJ, Algra A (2015). D-dimer for the exclusion of cerebral venous thrombosis: a meta-analysis of low risk patients with isolated headache. BMC Neurol, 15:118. 26215857 10.1186/s12883-015-0389-y PMC4517419

[87] Hu Y, Meng R, Zhang X, Guo L, Li S, Wu Y, et al (2018). Serum neuron specific enolase may be a marker to predict the severity and outcome of cerebral venous thrombosis. J Neurol, 265:46-51. 29128928 10.1007/s00415-017-8659-9

[88] Patel SI, Obeid H, Matti L, Ramakrishna H, Shamoun FE (2015). Cerebral Venous Thrombosis: Current and Newer Anticoagulant Treatment Options. Neurologist, 20:80-88. 26566039 10.1097/NRL.0000000000000049

[89] Covut F, Kewan T, Perez O, Flores M, Haddad A, Daw H (2019). Apixaban and rivaroxaban in patients with cerebral venous thrombosis. Thromb Res, 173:77-78. 30481600 10.1016/j.thromres.2018.11.018

[90] Shankar Iyer R, Tcr R, Akhtar S, Muthukalathi K, Kumar P, Muthukumar K (2018). Is it safe to treat cerebral venous thrombosis with oral rivaroxaban without heparin? A preliminary study from 20 patients. Clin Neurol Neurosurg, 175:108-111. 30396036 10.1016/j.clineuro.2018.10.015

[91] Desai NR, Cornutt D (2019). Reversal agents for direct oral anticoagulants: considerations for hospital physicians and intensivists. Hosp Pract (1995), 47:113-122. 31317796 10.1080/21548331.2019.1643728

[92] Ding J, Zhou D, Hu Y, Elmadhoun O, Pan L, Ya J, et al (2018). The efficacy and safety of Batroxobin in combination with anticoagulation on cerebral venous sinus thrombosis. J Thromb Thrombolysis, 46:371-378. 30062617 10.1007/s11239-018-1718-y

[93] Ding JY, Pan LQ, Hu YY, Rajah GB, Zhou D, Bai CB, et al (2019). Batroxobin in combination with anticoagulation may promote venous sinus recanalization in cerebral venous thrombosis: A real-world experience. CNS Neurosci Ther. 10.1111/cns.13093 PMC6488911 30675757

[94] Salottolo K, Wagner J, Frei DF, Loy D, Bellon RJ, McCarthy K, et al (2017). Epidemiology, Endovascular Treatment, and Prognosis of Cerebral Venous Thrombosis: US Center Study of 152 Patients. J Am Heart Assoc, 6. 10.1161/JAHA.117.005480 PMC5669171 28611097

[95] Aguiar de Sousa D, Lucas Neto L, Canhao P, Ferro JM (2018). Recanalization in Cerebral Venous Thrombosis. Stroke, 49:1828-1835. 30021808 10.1161/STROKEAHA.118.022129

[96] Qureshi AI, Grigoryan M, Saleem MA, Aytac E, Wallery SS, Rodriguez GJ, et al (2018). Prolonged Microcatheter-Based Local Thrombolytic Infusion as a Salvage Treatment After Failed Endovascular Treatment for Cerebral Venous Thrombosis: A Multicenter Experience. Neurocrit Care, 29:54-61. 29484582 10.1007/s12028-018-0502-3

[97] Lee SK, Mokin M, Hetts SW, Fifi JT, Bousser MG, Fraser JF (2018). Current endovascular strategies for cerebral venous thrombosis: report of the SNIS Standards and Guidelines Committee. J Neurointerv Surg, 10:803-810. 29871990 10.1136/neurintsurg-2018-013973

[98] Mendes PD, Lopes C, Franca DM, Reis JC, Santos ACJ, Darwich RZ (2018). The Dilemma of Anticoagulating Patients with Cerebral Venous Thrombosis Who Underwent Decompressive Craniectomy. World Neurosurg, 114:168-171. 29574222 10.1016/j.wneu.2018.03.103

[99] Venkateswaran P, Sriganesh K, Chakrabarti D, Srinivas DB, Rao GSU (2018). Regional Cerebral Oxygen Saturation Changes After Decompressive Craniectomy for Malignant Cerebral Venous Thrombosis: A Prospective Cohort Study. J Neurosurg Anesthesiol. 10.1097/ANA.0000000000000498 29481444

[100] Mahale R, Mehta A, Varma RG, Hegde AS, Acharya PT, Srinivasa R (2017). Decompressive surgery in malignant cerebral venous sinus thrombosis: what predicts its outcome? J Thromb Thrombolysis, 43:530-539. 28299538 10.1007/s11239-017-1489-x

[101] Lechanoine F, Janot K, Herbreteau D, Maldonado IL, Velut S (2018). Surgical Thrombectomy Combined with Bilateral Decompressive Craniectomy in a Life-Threatening Case of Coma from Cerebral Venous Sinus Thrombosis: Case Report and Literature Review. World Neurosurg, 120:485-489. 30253994 10.1016/j.wneu.2018.09.083

[102] Higgins JN, Burnet NG, Schwindack CF, Waters A (2008). Severe brain edema caused by a meningioma obstructing cerebral venous outflow and treated with venous sinus stenting. Case report. J Neurosurg, 108:372-376. 18240938 10.3171/JNS/2008/108/2/0372

[103] Ganesan D, Higgins JN, Harrower T, Burnet NG, Sarkies NJ, Manford M, et al (2008). Stent placement for management of a small parasagittal meningioma. Technical note. J Neurosurg, 108:377-381. 18240939 10.3171/JNS/2008/108/2/0377

[104] Debernardi A, Quilici L, La Camera A, Boccardi E, Cenzato M (2018). Torcular Meningioma with Multi-Venous Sinus Invasion: Compensatory Drainage Veins and Surgical Strategy. World Neurosurg, 109:451-454. 29097333 10.1016/j.wneu.2017.10.120

[105] Mantovani A, Di Maio S, Ferreira MJ, Sekhar LN (2014). Management of meningiomas invading the major dural venous sinuses: operative technique, results, and potential benefit for higher grade tumors. World Neurosurg, 82:455-467. 23851229 10.1016/j.wneu.2013.06.024

[106] Riggeal BD, Bruce BB, Saindane AM, Ridha MA, Kelly LP, Newman NJ, et al (2013). Clinical course of idiopathic intracranial hypertension with transverse sinus stenosis. Neurology, 80:289-295. 23269597 10.1212/WNL.0b013e31827debd6 PMC3589184

[107] King JO, Mitchell PJ, Thomson KR, Tress BM (2002). Manometry combined with cervical puncture in idiopathic intracranial hypertension. Neurology, 58:26-30. 11781401 10.1212/wnl.58.1.26

[108] Leach JL, Meyer K, Jones BV, Tomsick TA (2008). Large arachnoid granulations involving the dorsal superior sagittal sinus: findings on MR imaging and MR venography. AJNR Am J Neuroradiol, 29:1335-1339. 18417601 10.3174/ajnr.A1093 PMC8119153

[109] Umeh R, Oskouian RJ, Loukas M, Tubbs RS (2017). Giant Arachnoid Granulation Associated with Anomalous Draining Vein: A Case Report. Cureus, 9:e1065. 28409066 10.7759/cureus.1065 PMC5375954

[110] Rodrigues JR, Santos GR (2017). Brain Herniation into Giant Arachnoid Granulation: An Unusual Case. Case Rep Radiol, 2017:8532074. 28392955 10.1155/2017/8532074 PMC5368369

[111] Leach JL, Jones BV, Tomsick TA, Stewart CA, Balko MG (1996). Normal appearance of arachnoid granulations on contrast-enhanced CT and MR of the brain: differentiation from dural sinus disease. AJNR Am J Neuroradiol, 17:1523-1532. 8883652 PMC8338735

[112] Mattle HP, Wentz KU, Edelman RR, Wallner B, Finn JP, Barnes P, et al (1991). Cerebral venography with MR. Radiology, 178:453-458. 1987608 10.1148/radiology.178.2.1987608

[113] Huston J, 3rd, Ehman RL (1993). Comparison of time-of-flight and phase-contrast MR neuroangiographic techniques. Radiographics, 13:5-19. 8426937 10.1148/radiographics.13.1.8426937

[114] Lim RP, Koktzoglou I (2015). Noncontrast magnetic resonance angiography: concepts and clinical applications. Radiol Clin North Am, 53:457-476. 25953284 10.1016/j.rcl.2014.12.003

[115] Mandeville ET, Ayata C, Zheng Y, Mandeville JB (2017). Translational MR Neuroimaging of Stroke and Recovery. Transl Stroke Res, 8:22-32. 27578048 10.1007/s12975-016-0497-z PMC5243530

[116] van den Bergh WM, van der Schaaf I, van Gijn J (2005). The spectrum of presentations of venous infarction caused by deep cerebral vein thrombosis. Neurology, 65:192-196. 16043785 10.1212/01.wnl.0000179677.84785.63

[117] Bousser MG (2000). Cerebral venous thrombosis: diagnosis and management. J Neurol, 247:252-258. 10836615 10.1007/s004150050579

[118] Hu HH, Campeau NG, Huston J 3rd, Kruger DG, Haider CR, Riederer SJ (2007). High-spatial-resolution contrast-enhanced MR angiography of the intracranial venous system with fourfold accelerated two-dimensional sensitivity encoding. Radiology, 243:853-861. 17446523 10.1148/radiol.2433060819 PMC2813572

[119] Casey SO, Alberico RA, Patel M, Jimenez JM, Ozsvath RR, Maguire WM, et al (1996). Cerebral CT venography. Radiology, 198:163-170. 8539371 10.1148/radiology.198.1.8539371

[120] Ozsvath RR, Casey SO, Lustrin ES, Alberico RA, Hassankhani A, Patel M (1997). Cerebral venography: comparison of CT and MR projection venography. AJR Am J Roentgenol, 169:1699-1707. 9393193 10.2214/ajr.169.6.9393193

[121] Liauw L, van Buchem MA, Spilt A, de Bruine FT, van den Berg R, Hermans J, et al (2000). MR angiography of the intracranial venous system. Radiology, 214:678-682. 10715029 10.1148/radiology.214.3.r00mr41678

[122] Vogl TJ, Bergman C, Villringer A, Einhaupl K, Lissner J, Felix R (1994). Dural sinus thrombosis: value of venous MR angiography for diagnosis and follow-up. AJR Am J Roentgenol, 162:1191-1198. 8166009 10.2214/ajr.162.5.8166009

[123] Zhou D, Ding JY, Ya JY, Pan LQ, Yan F, Yang Q, et al (2018). Understanding jugular venous outflow disturbance. CNS Neurosci Ther, 24:473-482. 29687619 10.1111/cns.12859 PMC6489808

[124] Wetzel SG, Kirsch E, Stock KW, Kolbe M, Kaim A, Radue EW (1999). Cerebral veins: comparative study of CT venography with intraarterial digital subtraction angiography. AJNR Am J Neuroradiol, 20:249-255. 10094346 PMC7056122

[125] Baumgartner RW, Gonner F, Arnold M, Muri RM (1997). Transtemporal power- and frequency-based color-coded duplex sonography of cerebral veins and sinuses. AJNR Am J Neuroradiol, 18:1771-1781. 9367330 PMC8338469

[126] Stolz E, Kaps M, Kern A, Babacan SS, Dorndorf W (1999). Transcranial color-coded duplex sonography of intracranial veins and sinuses in adults. Reference data from 130 volunteers. Stroke, 30:1070-1075. 10229746 10.1161/01.str.30.5.1070

[127] Stolz E, Babacan SS, Bodeker RH, Gerriets T, Kaps M (2001). Interobserver and intraobserver reliability of venous transcranial color-coded flow velocity measurements. J Neuroimaging, 11:385-392. 11677878 10.1111/j.1552-6569.2001.tb00067.x

[128] Valdueza JM, Hoffmann O, Doepp F, Lehmann R, Einhaupl KM (1998). Venous Doppler ultrasound assessment of the parasellar region. Cerebrovasc Dis, 8:113-117. 9548010 10.1159/000015828

[129] Markl M, Frydrychowicz A, Kozerke S, Hope M, Wieben O (2012). 4D flow MRI. J Magn Reson Imaging, 36:1015-1036. 23090914 10.1002/jmri.23632

[130] Stolz E, Kaps M, Dorndorf W (1999). Assessment of intracranial venous hemodynamics in normal individuals and patients with cerebral venous thrombosis. Stroke, 30:70-75. 9880391 10.1161/01.str.30.1.70

[131] Filippidis A, Kapsalaki E, Patramani G, Fountas KN (2009). Cerebral venous sinus thrombosis: review of the demographics, pathophysiology, current diagnosis, and treatment. Neurosurg Focus, 27:E3. 10.3171/2009.8.FOCUS09167 19877794

[132] Schuchardt F, Hennemuth A, Schroeder L, Meckel S, Markl M, Wehrum T, et al (2017). Acute Cerebral Venous Thrombosis: Three-Dimensional Visualization and Quantification of Hemodynamic Alterations Using 4-Dimensional Flow Magnetic Resonance Imaging. Stroke, 48:671-677. 28179559 10.1161/STROKEAHA.116.015102

[133] Ono Y, Abe K, Suzuki K, Iimura H, Sakai S, Uchiyama S, et al (2013). Usefulness of 4D-CTA in the detection of cerebral dural sinus occlusion or stenosis with collateral pathways. Neuroradiol J, 26:428-438. 24007731 10.1177/197140091302600408 PMC4202811

[134] Mokin M, Kan P, Abla AA, Kass-Hout T, Snyder KV, Levy EI, et al (2013). Intravascular ultrasound in the evaluation and management of cerebral venous disease. World Neurosurg, 80:655.e657-613. 10.1016/j.wneu.2012.04.004 22484769

[135] Yan F, Rajah G, Ding Y, Hua Y, Zhang H, Jiao L, et al (2019). Safety and efficacy of intravascular ultrasound as an adjunct to stenting for cerebral venous sinus stenosis-induced idiopathic intracranial hypertension: a pilot study. J Neurosurg:1-6. 10.3171/2018.11.JNS181885 30835685

[136] Levitt MR, Albuquerque FC, Gross BA, Moon K, Jadhav AP, Ducruet AF, et al (2017). Venous sinus stenting in patients without idiopathic intracranial hypertension. J Neurointerv Surg, 9:512-515. 27199383 10.1136/neurintsurg-2016-012405

[137] McGonigal A, Bone I, Teasdale E (2004). Resolution of transverse sinus stenosis in idiopathic intracranial hypertension after L-P shunt. Neurology, 62:514-515. 14872049 10.1212/wnl.62.3.514

[138] Zivadinov R (2013). Is there a link between the extracranial venous system and central nervous system pathology? BMC Med, 11:259. 24344725 10.1186/1741-7015-11-259 PMC3866248

[139] Zhou D, Ding J, Asmaro K, Pan L, Ya J, Yang Q, et al (2019). Clinical Characteristics and Neuroimaging Findings in Internal Jugular Venous Outflow Disturbance. Thromb Haemost. 10.1055/s-0038-1676815 30605919

[140] Khandekar AA, Kumbhalkar SD, Salkar HR, Parakkadavathu RT, Salkar RG (2003). Protein S deficiency presenting as deep vein thrombosis--a case report. Angiology, 54:605-608. 14565637 10.1177/000331970305400511

[141] Ishida A, Matsuo S, Niimura K, Yoshimoto H, Shiramizu H, Hori T (2011). Cervical spontaneous spinal epidural hematoma with internal jugular vein thrombosis. J Neurosurg Spine, 15:187-189. 21513425 10.3171/2011.3.SPINE10673

[142] Yoshimatsu H, Yamamoto T, Iida T (2015). Indocyanine green angiography for prediction of thrombosis in the internal jugular vein. Microsurgery, 35:469-473. 26331715 10.1002/micr.22460

[143] Dolic K, Marr K, Valnarov V, Dwyer MG, Carl E, Karmon Y, et al (2012). Intra- and extraluminal structural and functional venous anomalies in multiple sclerosis, as evidenced by 2 noninvasive imaging techniques. AJNR Am J Neuroradiol, 33:16-23. 22194367 10.3174/ajnr.A2877 PMC7966175

[144] Zivadinov R, Chung CP (2013). Potential involvement of the extracranial venous system in central nervous system disorders and aging. BMC Med, 11:260. 24344742 10.1186/1741-7015-11-260 PMC3866257

[145] Valdueza JM, von Munster T, Hoffman O, Schreiber S, Einhaupl KM (2000). Postural dependency of the cerebral venous outflow. Lancet, 355:200-201. 10.1016/s0140-6736(99)04804-7 10675123

[146] Doepp F, Schreiber SJ, von Munster T, Rademacher J, Klingebiel R, Valdueza JM (2004). How does the blood leave the brain? A systematic ultrasound analysis of cerebral venous drainage patterns. Neuroradiology, 46:565-570. 15258709 10.1007/s00234-004-1213-3

[147] Tobinick E, Vega CP (2006). The cerebrospinal venous system: anatomy, physiology, and clinical implications. MedGenMed, 8:53. 16915183

[148] Zamboni P, Galeotti R, Menegatti E, Malagoni AM, Tacconi G, Dall'Ara S, et al (2009). Chronic cerebrospinal venous insufficiency in patients with multiple sclerosis. J Neurol Neurosurg Psychiatry, 80:392-399. 19060024 10.1136/jnnp.2008.157164 PMC2647682

[149] Chung CP, Beggs C, Wang PN, Bergsland N, Shepherd S, Cheng CY, et al (2014). Jugular venous reflux and white matter abnormalities in Alzheimer's disease: a pilot study. J Alzheimers Dis, 39:601-609. 24217278 10.3233/JAD-131112

[150] Liu M, Xu H, Wang Y, Zhong Y, Xia S, Utriainen D, et al (2015). Patterns of chronic venous insufficiency in the dural sinuses and extracranial draining veins and their relationship with white matter hyperintensities for patients with Parkinson's disease. J Vasc Surg, 61:1511-1520.e1511. 24655749 10.1016/j.jvs.2014.02.021 PMC4169367

[151] Bruno A, Napolitano M, Califano L, Attanasio G, Giugliano V, Cavazzuti PP, et al (2017). The Prevalence of Chronic Cerebrospinal Venous Insufficiency in Meniere Disease: 24-Month Follow-up after Angioplasty. J Vasc Interv Radiol, 28:388-391. 28034701 10.1016/j.jvir.2016.10.019

[152] Han K, Hu HH, Chao AC, Chang FC, Chung CP, Hsu HY, et al (2019). Transient Global Amnesia Linked to Impairment of Brain Venous Drainage: An Ultrasound Investigation. Front Neurol, 10:67. 30804883 10.3389/fneur.2019.00067 PMC6370701

[153] Traboulsee AL, Machan L, Girard JM, Raymond J, Vosoughi R, Hardy BW, et al (2018). Safety and efficacy of venoplasty in MS: A randomized, double-blind, sham-controlled, phase II trial. Neurology. 10.1212/WNL.0000000000006423 PMC6207414 30266886

[154] Thibault P, Attia J, Oldmeadow C (2018). A prolonged antibiotic protocol to treat persistent Chlamydophila pneumoniae infection improves the extracranial venous circulation in multiple sclerosis. Phlebology, 33:397-406. 28583026 10.1177/0268355517712884

[155] Thibault PK (2019). Neck vein obstruction: Diagnosis and the role of chronic persistent Chlamydophila pneumoniae infection. Phlebology, 34:372-379. 30360684 10.1177/0268355518804379

[156] Thibault PK (2012). Multiple sclerosis: a chronic infective cerebrospinal venulitis? Phlebology, 27:207-218. 22240624 10.1258/phleb.2011.011068

[157] Schrauben EM, Kohn S, Macdonald J, Johnson KM, Kliewer M, Frost S, et al (2017). Four-dimensional flow magnetic resonance imaging and ultrasound assessment of cerebrospinal venous flow in multiple sclerosis patients and controls. J Cereb Blood Flow Metab, 37:1483-1493. 27364001 10.1177/0271678X16657345 PMC5453467

[158] Rahman MT, Sethi SK, Utriainen DT, Hewett JJ, Haacke EM (2013). A comparative study of magnetic resonance venography techniques for the evaluation of the internal jugular veins in multiple sclerosis patients. Magn Reson Imaging, 31:1668-1676. 23850076 10.1016/j.mri.2013.05.012 PMC3932561

[159] Wattjes MP, van Oosten BW, de Graaf WL, Seewann A, Bot JC, van den Berg R, et al (2011). No association of abnormal cranial venous drainage with multiple sclerosis: a magnetic resonance venography and flow-quantification study. J Neurol Neurosurg Psychiatry, 82:429-435. 20980483 10.1136/jnnp.2010.223479

[160] McAuliffe W, Kermode AG (2013). Mystery of chronic cerebrospinal venous insufficiency: identical venographic and ultrasound findings in patients with MS and controls. AJNR Am J Neuroradiol, 34:1370-1374. 23370468 10.3174/ajnr.A3390 PMC8051509

[161] Kantarci F, Albayram S, Demirci NO, Esenkaya A, Uluduz D, Uysal O, et al (2012). Chronic cerebrospinal venous insufficiency: does ultrasound really distinguish multiple sclerosis subjects from healthy controls? Eur Radiol, 22:970-979. 22124776 10.1007/s00330-011-2338-5

[162] Thibault P, Lewis W, Niblett S (2015). Objective duplex ultrasound evaluation of the extracranial circulation in multiple sclerosis patients undergoing venoplasty of internal jugular vein stenoses: a pilot study. Phlebology, 30:98-104. 10.1177/0268355513515473 24321823

[163] Karmon Y, Zivadinov R, Weinstock-Guttman B, Marr K, Valnarov V, Dolic K, et al (2013). Comparison of intravascular ultrasound with conventional venography for detection of extracranial venous abnormalities indicative of chronic cerebrospinal venous insufficiency. J Vasc Interv Radiol, 24:1487-1498.e1481. 23953830 10.1016/j.jvir.2013.06.012

[164] Scalise F, Farina M, Manfredi M, Auguadro C, Novelli E (2013). Assessment of jugular endovascular malformations in chronic cerebrospinal venous insufficiency: colour-Doppler scanning and catheter venography compared with intravascular ultrasound. Phlebology, 28:409-417. 23155132 10.1258/phleb.2012.012079

[165] Veroux P, Giaquinta A, Perricone D, Lupo L, Gentile F, Virgilio C, et al (2013). Internal jugular veins out flow in patients with multiple sclerosis:a catheter venography study. J Vasc Interv Radiol, 24:1790-1797. 24409471 10.1016/j.jvir.2013.08.024

[166] Zivadinov R, Karmon Y, Dolic K, Hagemeier J, Marr K, Valnarov V, et al (2013). Multimodal noninvasive and invasive imaging of extracranial venous abnormalities indicative of CCSVI: results of the PREMiSe pilot study. BMC Neurol, 13:151. 24139135 10.1186/1471-2377-13-151 PMC4015359

[167] Traboulsee AL, Knox KB, Machan L, Zhao Y, Yee I, Rauscher A, et al (2014). Prevalence of extracranial venous narrowing on catheter venography in people with multiple sclerosis, their siblings, and unrelated healthy controls: a blinded, case-control study. Lancet, 383:138-145. 24119384 10.1016/S0140-6736(13)61747-X

[168] Lee BB, Baumgartner I, Berlien P, Bianchini G, Burrows P, Gloviczki P, et al (2015). Diagnosis and Treatment of Venous Malformations. Consensus Document of the International Union of Phlebology (IUP): updated 2013. Int Angiol, 34:97-149. 24566499

[169] Ferro JM, Bousser MG, Canhao P, Coutinho JM, Crassard I, Dentali F, et al (2017). European Stroke Organization guideline for the diagnosis and treatment of cerebral venous thrombosis - endorsed by the European Academy of Neurology. Eur J Neurol, 24:1203-1213. 28833980 10.1111/ene.13381

[170] Gianesini S, Menegatti E, Mascoli F, Salvi F, Bastianello S, Zamboni P (2014). The omohyoid muscle entrapment of the internal jugular vein. A still unclear pathogenetic mechanism. Phlebology, 29:632-635. 23761870 10.1177/0268355513489549

[171] Rai R, Ranade A, Nayak S, Vadgaonkar R, Mangala P, Krishnamurthy A (2008). A study of anatomical variability of the omohyoid muscle and its clinical relevance. Clinics (Sao Paulo), 63:521-524. 18719765 10.1590/S1807-59322008000400018 PMC2664130

[172] Zamboni P, Galeotti R, Menegatti E, Malagoni AM, Gianesini S, Bartolomei I, et al (2009). A prospective open-label study of endovascular treatment of chronic cerebrospinal venous insufficiency. J Vasc Surg, 50:1348-1358.e1341-1343. 19958985 10.1016/j.jvs.2009.07.096

[173] Zamboni P, Menegatti E, Cittanti C, Sisini F, Gianesini S, Salvi F, et al (2016). Fixing the jugular flow reduces ventricle volume and improves brain perfusion. J Vasc Surg Venous Lymphat Disord, 4:434-445. 27638998 10.1016/j.jvsv.2016.06.006

[174] Kazibudzki M, Latacz P, Ludyga T, Simka M (2016). Efficacy and safety of cutting balloons for the treatment of obstructive lesions in the internal jugular veins. J Cardiovasc Surg (Torino), 57:514-518. 24153192

[175] Lupattelli T, Bellagamba G, Righi E, Di Donna V, Flaishman I, Fazioli R, et al (2013). Feasibility and safety of endovascular treatment for chronic cerebrospinal venous insufficiency in patients with multiple sclerosis. J Vasc Surg, 58:1609-1618. 23948669 10.1016/j.jvs.2013.05.108

[176] Zamboni P, Tesio L, Galimberti S, Massacesi L, Salvi F, D'Alessandro R, et al (2018). Efficacy and Safety of Extracranial Vein Angioplasty in Multiple Sclerosis: A Randomized Clinical Trial. JAMA Neurol, 75:35-43. 29150995 10.1001/jamaneurol.2017.3825 PMC5833494

[177] Traboulsee AL, Machan L, Girard JM, Raymond J, Vosoughi R, Hardy BW, et al (2018). Safety and efficacy of venoplasty in MS: A randomized, double-blind, sham-controlled phase II trial. Neurology, 91:e1660-e1668. 30266886 10.1212/WNL.0000000000006423 PMC6207414

[178] Siddiqui AH, Zivadinov R, Benedict RH, Karmon Y, Yu J, Hartney ML, et al (2014). Prospective randomized trial of venous angioplasty in MS (PREMiSe). Neurology, 83:441-449. 24975855 10.1212/WNL.0000000000000638 PMC4132574

[179] Ghezzi A, Annovazzi P, Cocco E, Coarelli G, Lugaresi A, Rovaris M, et al (2013). Endovascular treatment of CCSVI in patients with multiple sclerosis: clinical outcome of 462 cases. Neurol Sci, 34:1633-1637. 23354606 10.1007/s10072-013-1300-5

[180] Ghezzi A, Annovazzi P, Amato MP, Capello E, Cavalla P, Cocco E, et al (2013). Adverse events after endovascular treatment of chronic cerebro-spinal venous insufficiency (CCSVI) in patients with multiple sclerosis. Mult Scler, 19:961-963. 23380649 10.1177/1352458513475491

[181] Hayreh SS (2016). Pathogenesis of optic disc edema in raised intracranial pressure. Prog Retin Eye Res, 50:108-144. 26453995 10.1016/j.preteyeres.2015.10.001 PMC4698254

